# Transcriptome sequencing and annotation of the polychaete *Hermodice carunculata* (Annelida, Amphinomidae)

**DOI:** 10.1186/s12864-015-1565-6

**Published:** 2015-06-10

**Authors:** Shaadi Mehr, Aida Verdes, Rob DeSalle, John Sparks, Vincent Pieribone, David F Gruber

**Affiliations:** Biological Science Department, State University of New York, College at Old Westbury, Old Westbury, NY, 11568 USA; American Museum of Natural History, Sackler Institute for Comparative Genomics, Central Park W at 79th St, New York, NY 10024 USA; Baruch College and The Graduate Center, Department of Natural Sciences, City University of New York, New York, NY 10010 USA; American Museum of Natural History, Department of Ichthyology, American Museum of Natural History, Division of Vertebrate Zoology, New York, NY 10024 USA; John B. Pierce Laboratory, Cellular and Molecular Physiology, Yale University, New Haven, CT 06519 USA

**Keywords:** Next-generation sequencing, *Hermodice carunculata*, Polychaete, Molecular phylogenetics, *de novo* assembly, Functional annotation

## Abstract

**Background:**

The amphinomid polychaete *Hermodice carunculata* is a cosmopolitan and ecologically important omnivore in coral reef ecosystems, preying on a diverse suite of reef organisms and potentially acting as a vector for coral disease. While amphinomids are a key group for determining the root of the Annelida, their phylogenetic position has been difficult to resolve, and their publically available genomic data was scarce.

**Results:**

We performed deep transcriptome sequencing (Illumina HiSeq) and profiling on *Hermodice carunculata* collected in the Western Atlantic Ocean. We focused this study on 58,454 predicted Open Reading Frames (ORFs) of genes longer than 200 amino acids for our homology search, and Gene Ontology (GO) terms and InterPro IDs were assigned to 32,500 of these ORFs. We used this *de novo* assembled transcriptome to recover major signaling pathways and housekeeping genes. We also identify a suite of *H. carunculata* genes related to reproduction and immune response.

**Conclusions:**

We provide a comprehensive catalogue of annotated genes for *Hermodice carunculata* and expand the knowledge of reproduction and immune response genes in annelids, in general. Overall, this study vastly expands the available genomic data for *H. carunculata,* of which previously consisted of only 279 nucleotide sequences in NCBI. This underscores the utility of Illumina sequencing for *de novo* transcriptome assembly in non-model organisms as a cost-effective and efficient tool for gene discovery and downstream applications, such as phylogenetic analysis and gene expression profiling.

**Electronic supplementary material:**

The online version of this article (doi:10.1186/s12864-015-1565-6) contains supplementary material, which is available to authorized users.

## Background

The amphinomid polychaete *Hermodice carunculata* (Annelida, Amphinomidae) is a cosmopolitan and ecologically important omnivore inhabiting coral reefs and other habitats throughout the Atlantic Ocean, including the Gulf of Mexico and the Caribbean Sea, as well as the Mediterranean and Red seas [1]. It is known to prey on a diverse suite of reef organisms such as zoanthids [2,3], scleractinian corals [4-7], milleporid hydrocorals [5,8], anemones [9] and gorgonians [5]. *Hermodice carunculata* is also a winter reservoir and spring-summer vector for the coral-bleaching pathogen *Vibrio shiloi* [10] and plays a complex and potentially ecologically important role in coral reef ecosystem health.

Amphinomidae is a well-delineated clade within aciculate polychaetes and it comprises approximately 200 described species from 25 genera [11-13]. Amphinomids are distributed worldwide and are known to inhabit intertidal, continental shelf and shallow reef communities, with a few species also recorded from the deep-sea [13]. The clade is primarily identified by a series of morphological apomorphies including nuchal organs situated on a caruncle, a ventral muscular eversible proboscis with thickened cuticle on circular lamellae, and calcareous chaetae [12,14]. Due to the lack of knowledge regarding their morphological variability (particularly within closely related genera), previous studies based mainly on morphology have failed to clarify the evolutionary history of the group, leading to taxonomic problems. In fact, several nominal species have been regarded as conspecifics, often without evaluation of molecular data, which might explain the common occurrence of cosmopolitan species within the clade [15]. Consequently, detailed revisions of species and even genera are needed [13], which incorporate molecular phylogenetic studies to clarify the affinities within the family [11,16]. Additionally, amphinomids are group with unclear phylogenetic position within Annelida as different studies find different evolutionary affinities for the group [16,17], but regarded as morphologically primitive and considered of prime interest for determining the root of the annelid Tree of Life [18]. However, the availability of genomic data in public databases for *Hermodice carunculata* and other amphinomid species is particularly scarce. Previous to this study, only 279 sequences were accessible in NCBI for *H. carunculata*.

Furthermore, the annelid *Hermodice carunculata* is a representative of the Lophotrochozoa, a clade of protostome bilaterian animals that comprises about half of the extant animal phyla, including Mollusca, the second most diverse phylum [19]. Annelids, in general, are of interest within lophotrochozoans because they are among the first coelomates [20] and polychaetes in particular, exhibit ancestral traits in body plan and embryonic development [20,21]. Nevertheless, polychaete annelids and lophotrochozoans have been heavily underrepresented in sequencing efforts, therefore, genomic resources for this key bilaterian clade are still relatively poor compared to the other two major bilaterian clades (Ecdysozoa and Deuterostomia) [21]. A more complete representation of taxa in the genomic databases is needed to better understand animal evolution and unravel the origins of organismal diversity, especially of crucial clades such as the Lophotrochozoa [21,22].

Here, we provide a *de novo* transcriptome assembly of *Hermodice carunculata*, a cosmopolitan Lophotrochozoan polychaete that inhabits coral reefs throughout the Atlantic Ocean. In this study we use the Illumina HiSeq platform to generate a cDNA library for *H. carunculata*. These Next-Generation Sequencing (NGS) libraries have an enormous sequencing depth and better effectiveness, producing at least 100 to 10,000 times higher throughput than classical Sanger sequencing [23]. This allows for the examination of thousands of transcripts from uncharacterized species and renders it useful for a wide range of biological applications including phylogenomics [24], regulatory gene discovery [25-28], molecular marker development [29], single nucleotide polymorphism (SNP) identification for trait adaptation [30,31], haplotype detection [32,33], and differential gene expression profiling [25,32]. In this study we provide a reference set of mRNA sequences for *H. carunculata,* which will facilitate annotation of the genome and future studies of polychaete evolution, systematics and functional genomics. We specifically focused on major signaling pathways and housekeeping genes, as well as genes related to reproduction and immune response, and we provide a comprehensive list of genes related to these key processes in the annelid *H. carunculata*.

## Results and discussion

### Sequencing and *de novo* assembly

Total RNA was extracted from the body-segment *H. carunculata.* The (A)^+^ RNA was isolated, sheered to smaller fragments, and reverse transcribed to make cDNA for sequencing with Hi-Seq Illumina 1000. Four hundred million paired-end strand-unspecific reads were obtained from one lane of one plate, generating 32.4 gigabase pairs (Gbp) of raw data that were uploaded to NCBI. Reads were checked for Phred-like quality scores above the Q30 level with FastQC [34]. We used the pipeline proposed in [35] to remove low quality reads for *de novo* assembly. HiSeq Illumina read sequences were assembled into 525,989 contigs longer than 200 bp, with an N50 of 1,095 and mean length of 722.30 bp, using ABySS 1.3.1 [36], followed by Blat (with default parameters) [37] for redundancy removal. A range of 8 k-mers (21–55) were used for ABySS runs, with the parameter q = 3 to trim low-quality bases from the ends of reads for each run. The final data set was filtered for contigs longer than 200 bp. Summary statistics for each k-mer assembly, as well as for the merged and redundant-removed set of contigs is outlined in Table 1. Paired-end reads and assembled contigs that do not contain ambiguous bases have been deposited into NCBI and can be downloaded at the NCBI Sequence Read Archive: http://www.ncbi.nlm.nih.gov/sra/SRX194586%5Baccn%5D.

**Table 1:** **Summary Statistics for individual and merged assemblies**

**Assembly**	**Number of transcripts > 200 bp**	**N50 bp**	**Mean length bp**	**Max length bp**	**Total number of bp**
K-mer 21	143,191	584	505.54	7,342	72,390,913
K-mer 25	160,583	771	605.87	13,382	97,292,569
K-mer 29	188,890	631	523.05	8,878	98,798,757
K-mer 35	225,756	689	551.61	11,724	124,529,844
K-mer 41	179,143	891	633.86	18,825	113,522,250
K-mer 45	171,154	983	667.66	24,711	114,273,429
K-mer 51	156,387	1,096	713.03	17,800	111,509,378
K-mer 55	144,565	1,160	740.32	14,922	107,023,822
Final	525,989	1,095	722.3	24,711	379,922,870
**Generated ORFs from Assembly**	**Number of ORFs >200 AA**	**N50 AA**	**Mean length AA**	**Max length AA**	**Total number of AA**
ORFs > 200AA	58,454	490	443.92	8,167	25,948,636

For each k-mer, data from AbySS is shown. The final assmbly is the result of merging the AbySS k-mer assemblies using BLAT to remove the redundancies. Predicted ORF’s longer than 200AA’s from this final contig set were used for annotation. K-mer = required length of overlap match between two reads in AbySS; N50 = length weigthed median contig length; bp = base pair; ORF = Open Reading Frame.

**Figure 1:**
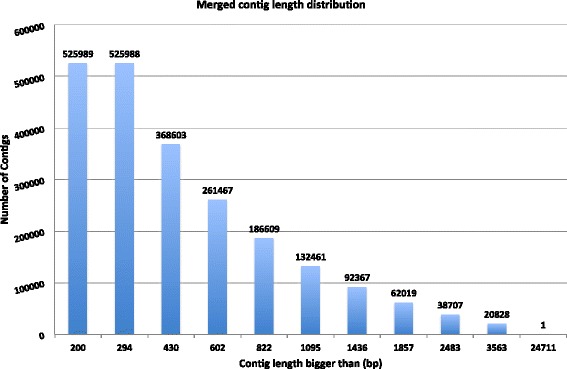
Assembled contig length distribution. Each number on top of each bar represents number of assembled contigs per length category.

Assemblies at higher k-mers (e.g. 41–55) had lower mean length and N50 than assemblies at lower k-mers (21–35) (Table 1). This is in agreement with other summary statistics of NGS reported *de novo* assembly data [38]. The lower N50 and mean in the final merged dataset, compared with k-mer 51 and k-mer 55, is due to addition of shorter sequences from lower k-mer assemblies. As outlined in Table 1, the N50 has changed from 584 in k-mer 21 to 1095 bp in the merged set of contigs, indicating an improvement in the assembly contig length. Although the majority of the contig length is between 200–600 bp, we obtained 20,828 contigs, with length greater than 3,563 bp (Figure 1). This result indicates that the data has a very high quality for further annotation. Lastly, the assembled sequences were deposited in Transcriptome Shotgun Assembly (TSA) at the NCBI.

A six frame translation (ORFs) from stop to stop for each assembled contig was generated using the EMBOSS package, version: 6.4.0.0 [39]. This file contained 58,454 predicted ORFs longer than 200 AA, with the N50 of 490 AA, and mean length of 443.92 AA.

### Comparative sequence similarity with other annelids

**Figure 2:**
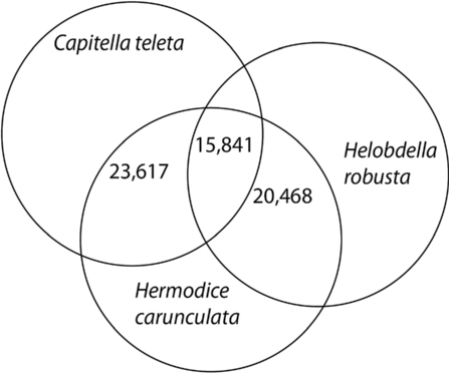
Venn diagram distribution of similarity search results for *Hermodice carunculata.* Based on 58,454 predicted Open Reading Frames (ORFs) of genes longer than 200 amino acids. The number of unique sequence-based annotation is the best sum of unique BlastP hits (E-value of 2e^−15^) from *Capitella teleta* and *Helobdella robusta* proteome, respectively.

For comparative annotation, all ORFs longer than 200 AA (58,454) were initially searched against two existing annelid genomic datasets, *Capitella teleta* (http://genome.jgi-psf.org/Capca1/Capca1.home.html) and *Helobdella robusta* (http://genome.jgi-psf.org/Helro1/Helro1.home.html); and subsequently against *Paramphinome jeffreysi* and *Eurythoe complanata*, using BlastP [40] with a significant E-value of 2e^**−15**^. Similarity search showed that 23,617 (40.5%) ORFs have similarity higher than 70% against *C. teleta,* while 20,468 (35%) ORFs have similarity higher than 70% against *H. robusta* (Figure 2). This indicates that the proportion of sequences with matches in the proteome of *C. teleta* is greater than the proportion of matches for *H. robusta*. This is expected, as *C. teleta* and *H. carunculata* are both polychaete annelids, as opposed to *H. robusta*, a leech (Clitellata). In total, 15,841 transcripts had a significant hit (70% length homology) in both datasets. Furthermore, 29,819 of these ORFs showed homology to *P. jeffreysi* and 36,033 to *E. complanata*. Of these ORFs, 23,441 were homologous to both *Paramphinome jeffreysi* and *Eurythoe complanata.* These shared sequences can be used for future genome annotation of both annelids and amphinomids, respectively (data available upon request).

### Functional annotation and characterization

One of the important aspects of mining the transcriptomic data is assigning function to individual transcripts. Functional annotation is an effective way to categorize genes into physiological classes to assist in understanding the large quantity of transcripts and for evaluating functional differences between subgroups of sequences. These data provide a tool for designing custom microarray experiments related to annotated functions [41]. Gene ontology (GO, http://www.geneontology.org) [42,43] is an extensive scheme for this purpose. This framework covers a wide biological scope, and with its directed acyclic graph (DAG) structure, it accounts for biological dependencies. In addition, programs such as InterProScan [44,45] provide an integrated platform for domain-based searches against databases such as PROSITE [46], PRINTS [47], Pfam [48], and SMART [49], in addition to others. Over the past few years, resources have been developed for automatic GO term and InterPro ID assignment to unknown sequences. Blast2GO [50] was utilized for functional annotation, visualization and its associated statistics.

As part of the Blast2GO pipeline, ORFs longer than 200 AA (58,454) were subjected to sequence homology search against the non-redundant protein database (NR) at NCBI, using BlastP (E 10–10, cutoff =55, GO weight = 5, HSP coverage = 0). Followed by mapping to collect GO terms, and assigning reliable information to each query sequence. Default values of Blast2GO annotation parameters were chosen to optimize the ratio between annotation accuracy and coverage [51]. This provided a framework for categorizing genes into functional annotation groups, namely biological process (sets of molecular events or operations with a defined beginning and end), molecular function (the primary activities of gene product at the molecular level, such as catalysis or binding), and cellular compartment. Furthermore, InterPro IDs (protein domain IDs) were assigned to sequences by running InterProScan (part of the Blast2Go pipeline).

**Figure 3:**
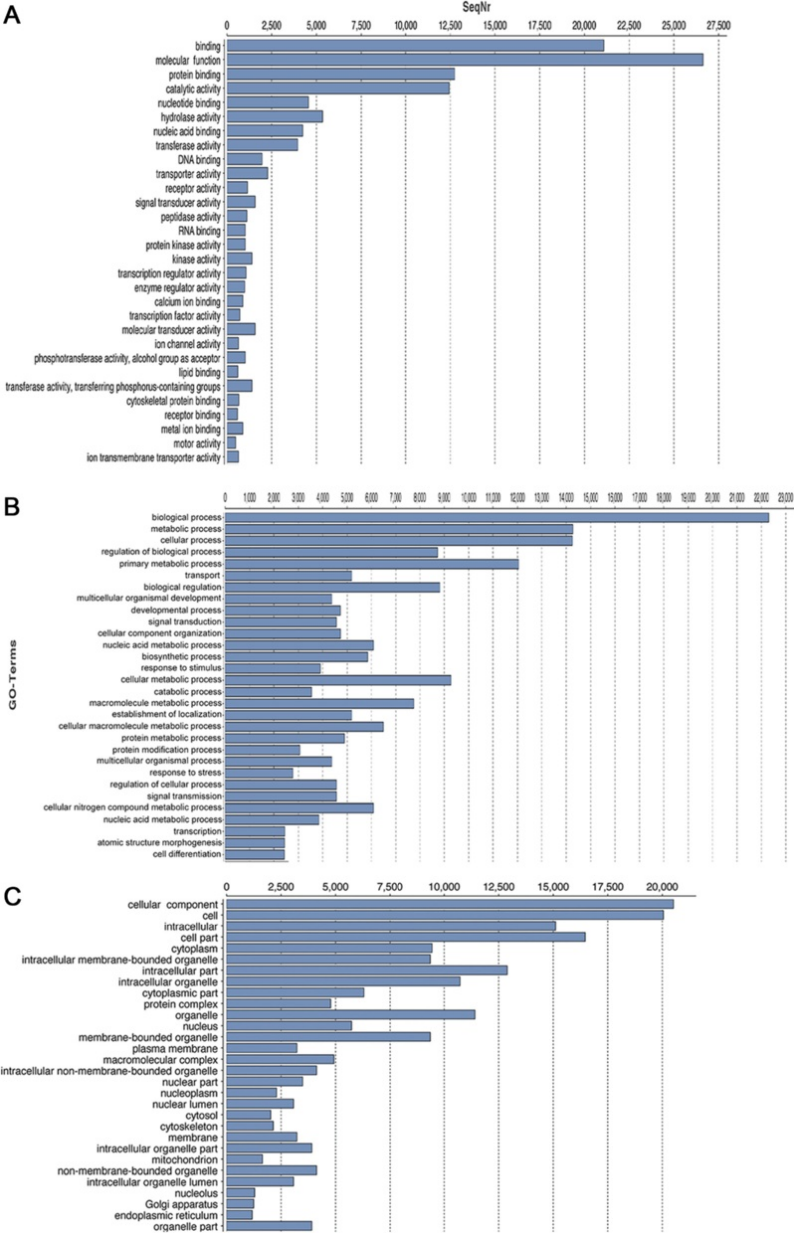
Functional annotation of *Hermodice carunculata* transcripts. The 30 most abundant GOslim terms based on **A** molecular function, **B** biological processes, **C** cellular component.

Out of 58,454 predicted ORFs, 55.6% (32,500) of the data contained definitive functional annotation. These sequences were classified into three categories (GOslim): biological process, cellular component and molecular function. The summary of classification of annotation is reported at Level 2 of GO Category. In the molecular function, the clusters relating to “binding” and “catalytic activity” were enriched (21,089 and 12,443, respectively) (Figure 3A). In the biological process classification, “metabolic process” with 14,272 sequences, “cellular processes” with 14,254 sequences, and “biological regulation” with 8,818 sequences were large compared to “regulation of anatomical structure size” and “cell growth” with about 200 sequences each (Figure 3B). This is expected, as these data are not collected from a developmental stage with high rate of divisions. In the cellular component category, the cluster size of “cell” with 20,053 sequences and “organelle” with 11,413 sequences were highly represented compared to “microbody” or “extracellular matrix” with less than 100 sequences each (Figure 3C). This pattern is very similar to a recent analysis of *Lymnae stagnalis* (pond snail) transcriptome functional annotation [26].

**Figure 4:**
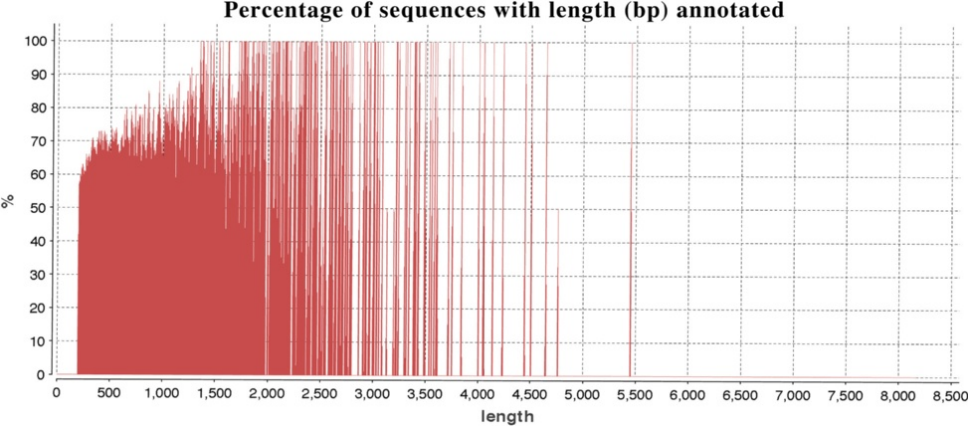
Percentage of functionally annotated transcripts relative to their length.

In terms of length distribution of annotated sequences, 70% to 90% of the sequences with length ranging from 200 AA to 1,500 AA were functionally annotated, while 100% of the sequences with length between 1,500 AA to 3,500 AA had a GO term assigned to them (Figure 4). This result indicates that longer sequences have a higher rate of annotation than shorter sequences. The annotated sequences and a table representing sequence IDs with their assigned GO terms and InterPro IDs and enzyme codes are reported (Additional file 1).

### Identification of candidate genes and potential phylogenetic markers

#### Signaling pathway and housekeeping genes

We identified 21 homologs of housekeeping genes belonging to CAT, MAT, PFK, ATP Synthase and 4,450 homologs of signaling pathways belonging to Activin, Deltex, DPP, Fringe, Jagged, Notch, Notch2, SMAD, TGF- β; (Additional file 2: Table S1). Riesgo and colleagues [52], in their analysis of ten transcriptomes of newly sequenced invertebrates, found similar homologs in mollusk and annelid transcriptomes.

#### Immune response genes

We identified 172 orthologous sequences of 37 genes involved in immune response (Additional file 2: Table S1), including caspase, interleukin, toll-like receptors, IRF genes, ficolin, antistasin and angiopoietin among others.

#### Reproduction genes

We identified 46 homologous sequences to 17 genes in>volved in reproduction, including attractin, vasa, germ cell-less, piwi, smaug, nanos, zona pellucida, spermatogenesis-associated proteins and zonadhesin (Additional file 2: Table S1).

#### Potential phylogenetic markers

Using reciprocal BLAST searches between the *Hermodice carunculata* transcriptome and publicly available sequences, we have identified putative *H. carunculata* homologues of genes that have been previously used as phylogenetic markers in Annelida but were unavailable for *H. carunculata* and amphinomids in general, with a few exceptions. We identified 900 homologous sequences of EF-1α, 101 homologous to H3, 7 homologous to CytB, and 400 homologous to U2 snRNA. We chose the longest sequence in each category for downstream phylogenetic analysis. The alignment of each of these sequences, along with the five best hits retrieved by BLAST from the NCBI database, are available in the supplementary materials (Additional files 3, 4, 5 and 6). Sequences were deposited in GeneBank.

#### Light production genes

**Figure 5:**
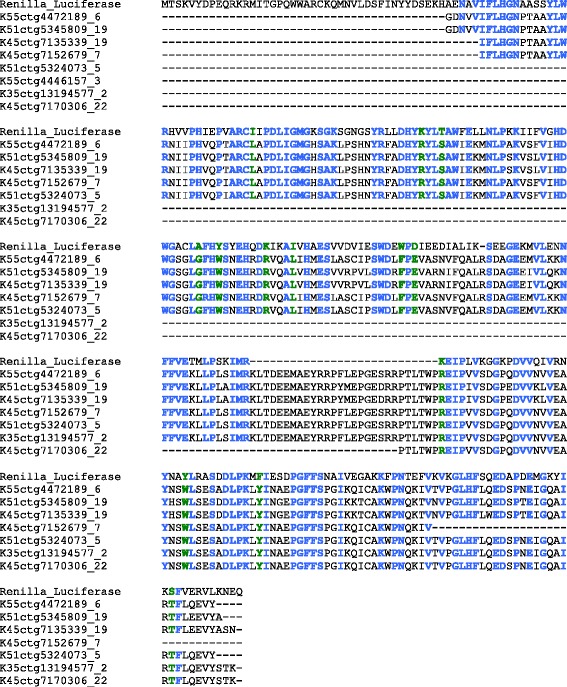
Overlapping region of amino acid sequence alignment of homologous proteins sequences to luciferase from the sea pansy, *Renilla* sp.

A search for sequence homology in the transcriptome of *Hermodice carunculata* against 182 known bioluminescent-related proteins, such as the photoproteins Obelin, Aequorin, and other luciferases, found eight sequence transcripts with an average of 44.9% homology to the luciferase protein of the phylogenetically distant sea pansy *Renilla reniformis* (Cnidaria, Renillidae). An alignment of the *H. carunculata* putative luciferase with *Renilla* luciferase is generated (Figure 5) and the corresponding cDNA sequences are included (Additional file 7).

### *In silico* quantification of the *hermodice carunculata* transcriptome

In order to identify poor quality and potentially misassembled transcripts, reads were mapped back onto the non-redundant set of transcripts [53]. The number of reads corresponding to each transcript ranged from 2 to 9000 with an average of 1,644 reads, indicating a wide range of expression (Additional file 8). This indicates that very low expressed transcripts were represented in our dataset. Furthermore, we analyzed the coverage of the functionally annotated transcripts. The minimum coverage was 2 FPKM and maximum was 20,000 FPKM. Among these, 400 transcripts had a mean coverage less than 3, or gaps were removed from dataset (Table 2).

**Table 2:** **Summary statistics of read counts and coverage**

**Total number of reads**	**426,555,924**
Number of read used reads for assembly	141,684,860 (33.22%)
Number of unused reads	28,4871,064 (66.78%)
Number of non-redundant transcripts (>200 bp)	525,989
Number of non-redundant trasncripts with back-aligned reads (>200 bp)	525,939
Number of transcripts with coverage fpkm >1	176,412
Number of transcripts with coverage fpkm >5	49,690
Average coverage for contigs from filtered dataset 2 (fpmk)	15.279
Average number of reads mapped per contig (with coverage fpkm >5)	1644

bp = base pair; fpkm = paired-reads per kilobase per million; contig = contiguous overlapping sequence read from assembly.

## Conclusions

Relying on Next Generation Sequencing techniques and a thorough bioinformatics pipeline we have generated a comprehensive list of major signaling pathways, housekeeping genes, and genes related to reproduction and immune response in a representative of the Lophotrochozoa, the polychaete annelid *Hermodice carunculata*, whose phylogenetic placement within Annelida has been difficult to resolve. Major signaling pathways are highly evolutionarily conserved across Metazoa and play an important role during embryonic and adult development, regulating many fundamental cellular processes such as proliferation, stem cell maintenance, differentiation, migration or apoptosis [54]. In addition, some genes such as those involved in Notch signaling might have a role in segment formation and adult regeneration in polychaetes [55]. Housekeeping genes are required for the maintenance of essential basal cellular functions and consequently, under normal conditions, they are expressed in all cells regardless of tissue type or developmental stage [56]. They are especially interesting because they represent the minimal set of genes required to sustain life and they can be used as comparative controls for experimental and computational studies [56], for example, to assess the suitability of transcriptome datasets for gene discovery [52]. Immune response genes are also of great concern especially among invertebrates because they represent an early model of the more highly evolved innate immune system of vertebrates [57]. Knowledge of the invertebrate immune system is based mainly in two ecdysozoan model organisms, *Drosophila melanogaster* and *Caenorabditis elegans*, and although Lophotrochozoan systems show some distinct differences [58], studies focusing on this group are very limited. Lastly, characterization of the reproductive genes of polychaetes is of interest as they exhibit an astonishing diversity of reproductive strategies, including both sexual and asexual reproduction, and range from spawning and external fertilization to brooding or viviparism, often involving marked morphological, physiological and behavioral modifications [12]. For example, some amphinomids such as *Eurythoe complanata* or *Cryptonome conclava* exhibit both sexual and asexual reproduction, the latter accomplished by architomic scissiparity: the body fragments in two or more parts which regenerate head, tail or both [13,59].

**Figure 6:**
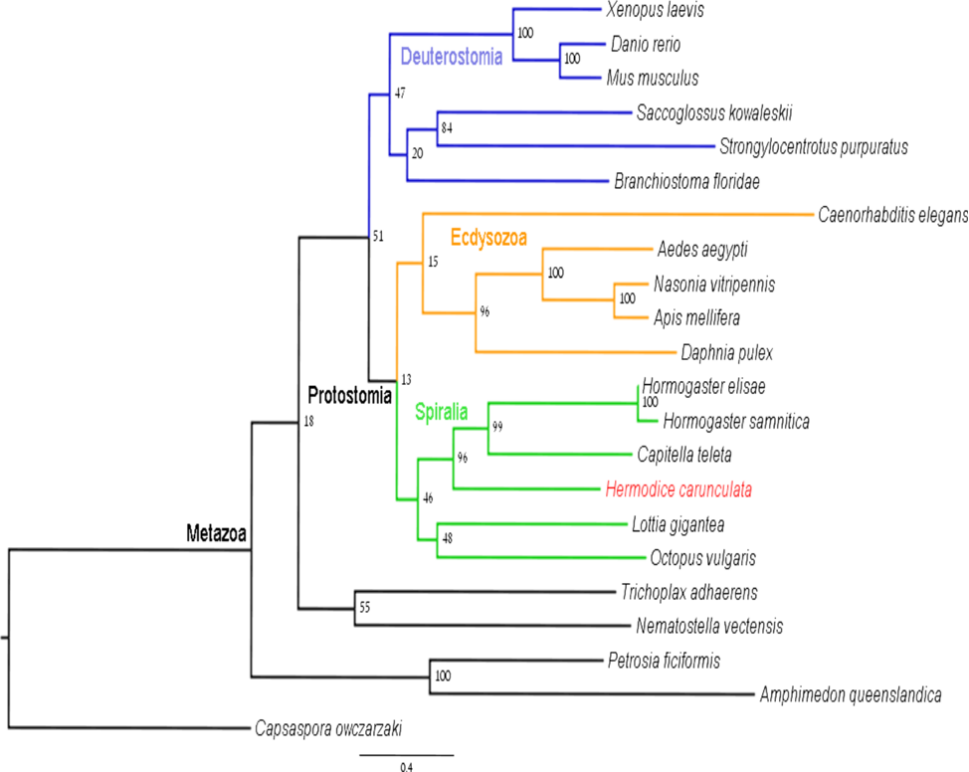
Maximum likelihood tree of 21 Attractin proteins and one newly identified attractin sequence from *Hermodice carunculata*. The newly identified attractin is colored red.

Sex pheromones have been postulated to drive cryptic speciation in oligochaetes [60]. Within polychaetes, there are several species known to use pheromones to attract the opposite sex and to control the release of gametes, such as the scale worm *Harmothoe imbricata* [61], the rag worms *Nereis succinea* and *Platynereis dumerilii* and the lugworm *Arenicola marina* [62]. The sex pheromone attractin has been suggested by previous authors as a potential phylogenetic marker [60]. As part of our annotation pipeline, we have identified seven sequences homologous to attractin in the transcriptome of *Hermodice carunculata*. A phylogenetic analysis was performed to evaluate the potential of the *H. carunculata* attractin protein as a reliable phylogenetic marker for polychaete systematics and evolutionary studies. Our analysis corroborates results by previous authors [60] suggesting that attractin represents an effective phylogenetic marker, recovering deep metazoan relationships (Figure 6; Additional file 9) and important clades such as Bilateria, its split into Deuterostomia and Protostomia, and the subdivision of the latter in Ecdysozoa and Spiralia (Lophotrochozoa). Attractin also recovers Annelida as a monophyletic group (Figure 6).

Several so-called cosmopolitan species within amphinomids have proven to comprise various cryptic species [1]. *Hermodice carunculata* has a widespread distribution and has been reported throughout the Atlantic Ocean, Caribbean, Mediterranean and Red Sea [63,64]. Despite its widespread distribution, its representation in NCBI consisted of only 359 nucleotide sequences and only a handful of studies have examined genetic aspects of *H. carunculata*. For example, in a species delineation study, two mitochondrial genes (COI and 16S rDNA) and the internal transcribed spacer 1 (ITS1) were used to test for cryptic speciation in *H. carunculata* [1]. This analysis showed that genetic divergence is low among samples across the Atlantic Ocean, and these particular three genes do not reflect any genetic basis for the observed morphological differences (e.g., variable filament abundance) among populations. Therefore, identification of informative loci for phylogeographic application is necessary. However, a different study using COI sequences has found that *Eurythoe complanata* represents a complex of three genetically distinct and morphologically indistinguishable lineages inhabiting the Atlantic and Pacific Oceans. Also, the deep-sea genus *Archinome* has been shown to comprise four genetically distinct lineages with no apparent morphological differences [65]. Therefore, the *de novo* assembled transcriptome presented herein for *Hermodice carunculata*, can also be used to develop additional molecular phylogenetic markers to aid forthcoming studies of species boundaries and evolutionary relationships within Amphinomidae. Furthermore, amphinomids are a morphologically plesiomorphic group of annelids, considered as a highly important taxon for reconstructing relationships at the base of the annelid tree [18]. Thus, the vast amount of molecular data provided herein can also help to elucidate the basal relationships of Annelida.

**Figure 7:**
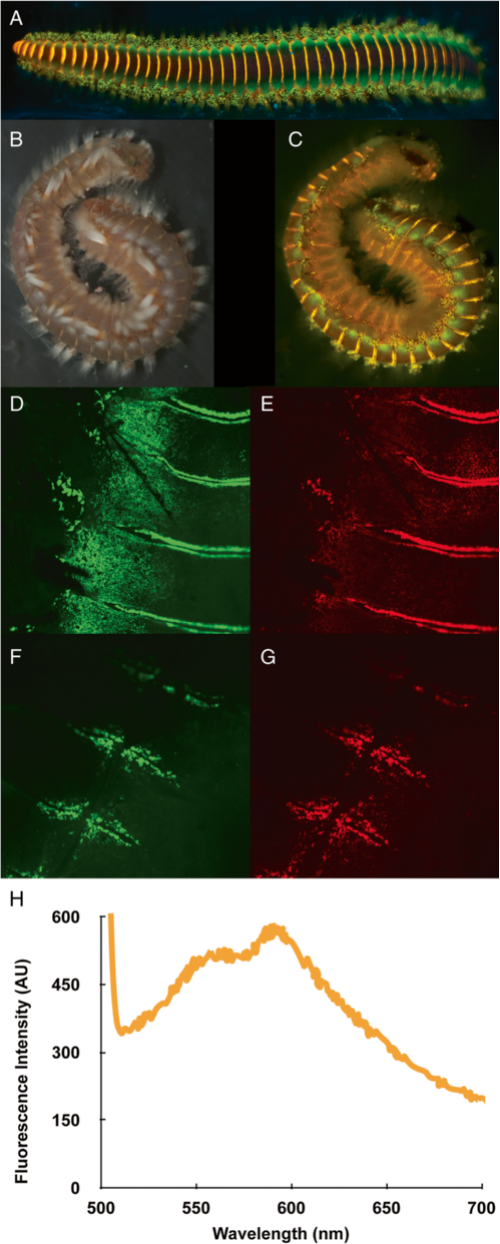
Fluorescent macro image of *Hermodice carunculata* using 450–500 nm excitation and 514 nm LP emission **(A)**; white light image **(B)**; and fluorescent macro comparison (using 450-500 nm excitation and 514nmLP emission) **(C)**; confocal images **(D-** **G)** obtained with a Olympus Fluoview FV1000 (Olympus, Japan) confocal laser scanning microscope using an Olympus LUMFL 60×/1.10 W objective (excitation 488 nm wavelength Ar-laser was used), illustrating distrubution of green and red fluorescence; **(H)** Emission spectra using an Ocean Optics USB2000+ miniature spectrometer (Dunedin, FL) equipped with a hand-held fiber optic probe (Ocean Optics ZFQ-12135).

Within annelid polychaetes there are a number of bioluminescent species distributed in various families such as Acrocirridae (*Swima*), Chaetopteridae (*Chaetopterus*), Flabelligeridae (*Poeobius*, *Flota*), Polynoidae (*Harmothoe*, *Polynoe*), Syllidae (*Odontosyllis*, *Eusyllis*, *Pionosyllis*), Terebellidae (*Polycirrus*, *Thelepus*) and Tomopteridae (*Tomopteris*) [66]. To date, no bioluminescent protein sequence has been reported from this phylum, but we do report homologous sequences of a luciferase protein (Figure 5). The fact that the putative *Hermodice carunculata* luciferase shows highest homology to the luciferase of a phylogenetically distant cnidarian (*Renilla reniformis*) can probably be attributable to the lack of publicly available luciferase sequences from more closely related organisms. The transcriptomic dataset presented herein can greatly help identify and characterize this putative photoprotein and facilitate future studies investigating the genetic and biochemical basis of light production in annelids. In addition, we report both green and red biofluorescence in *Hermodice carunculata,* yet the search of the genome showed no homology to any known fluorescent protein species (Figure 7).

An additional recent approach in estimating more accurate intergeneric and intrageneric level relationships utilizes conserved blocks of homologous sequences shared between genomic regions of multiple species [67]. Our data provides a complementary resource for this kind of application in the future. Also, the annotation of the genomes is reliant on transcriptome data for the exon intron boundary delimitation. Our data provide a base for future genomic and ecological research on *Hermodice carunculata*, as well as a resource to understand the natural history of polychaetes and the evolution of annelids in general.

## Methods

### Sample collection

Research, collecting and export permits were obtained from the government of the Bahamas while working out of the Perry Institute for Marine Science on Lee Stocking Island during a December 2011 expedition. The sample was collected by scientific divers D. Gruber, J. Sparks and M. Lombardi from Norman’s Pond Cay Cave, Norman’s Pond Cay, Exumas, Bahamas (GPS N 23 47.181, W 076 08.428). The cave’s entrance is a 2 m by 8 m sinkhole located just above high tide level and the cave is approximately 50 m linear and to a depth of 40 m. Divers explored the walls of the cavern zone using compact LED lights for cryptic invertebrate specimens. The *Hermodice carunculata* specimen was collected 30 m within the cave, transported back to the field station where it was frozen in liquid nitrogen less than two hours following collection.

### RNA extraction and transcriptome sequencing

Total RNA was extracted from dissected tail muscles. The muscle tissue was homogenized in TriZol reagent (Life Technologies, NY) and the total RNA was precipitated with isopropanol and dissolved in ddH_2_O. The quality of RNA was assessed on a 2100 Bioanalyzer and with agarose gel electrophoresis. The total RNA was pooled for Library preparation. Libraries were prepared using a HiSeq RNA sample preparation kit (Illumina Inc, San Diego, CA) according to the manufacturer’s instructions. One lane was multiplexed for four samples and was sequenced as 80-bp PE reads. FASTQ file generation was performed by CASAVA version 1.8.2 (Illumina).

### *De novo* assembly

All the assemblies were performed on a server with 50 cores and 250 GB random access memory. Obtained reads were *de novo* assembled, using ABySS [36] followed by Blat version: 34x12 [37], according to the proposed pipeline for merge and redundancy removal [35] in contigs generated by ABySS. In order to recover high and low expressed transcripts, a range of k-mers (21–55) was used prior to merge with Blat.

### Phylogenetic analysis

Sequences for the sex pheromone attractin were downloaded from GenBank (accession number generation in progress) and aligned with the *Hermodice carunculata* translated sequence using MUSCLE in SEAVIEW 4.3.0 [68]. A phylogenetic analysis using amino acid sequences was conducted with RAxML ver. 7.7.1 [69] using the maximum likelihood optimality criterion with a JTT amino acid substitution model. Support values were estimated using a rapid bootstrap algorithm with 1,000 replicates. The protozoan symbiont *Capsaspora owczarzaki* was specified as the outgroup.

### Functional annotation

Gene ontology (GO) terms and InterPro IDs were assigned to ORF sequences longer than 200 AA, using Blast2GO [50].

### Availability of supporting data

*Hermodice carunculata* paired-end reads and assembled contigs can be downloaded at the NCBI Sequence Read Archive: http://www.ncbi.nlm.nih.gov/sra/SRX194586%5Baccn%5D. We have also made available at LabArchives (https://mynotebook.labarchives.com/share/smehr/MjAuOHw4NTE4MS8xNi9UcmVlTm9kZS8yNzE4MjI2NjQ1fDUyLjg=): 1) a Fasta file of homologous of contigs shared between *Capitella teleta*, *Helobdella robusta* and *Hermodice carunculata*; 2) a Fasta file of homologous contigs shared between *Eurythoe complanata*, *Paramphinome jeffreysii* and *Hermodice carunculata*; and 3) the functionally annotated Open Reading Frames generated from the *Hermodice carunculata* transcriptome.

## Additional files

Additional file 1:
**Table of assigned Go terms and InterPro IDs for Open Reading Frame generated from** ***Hermodice carunculata*** **transcriptome.**
Additional file 2: Table S1. List of *Hermodice carunculata* reproduction and immune response genes. The specific gene, number of found contigs and the contig identification tag are included. Additional file 3:
**Fasta file of annotated EF-α expressed isoforms.**
Additional file 4:
**Fasta file of annotated Histon expressed isoforms.**
Additional file 5:
**Fasta file of annotated Cytochrome B expressed isoforms.**
Additional file 6:
**Fasta file of annotated U2 snRNA expressed isoforms.**
Additional file 7:
**Fasta file of annotated luciferases expressed isoforms.**
Additional file 8:
**RPKM of the assembled contigs.**
Additional file 9:
**Multiple Sequence Alignment of annotated attractin protein from** ***Hermodice carunculata*** **along with 21 other attractin sequences from other species.**
