# Supplementary material for: Transcriptome sequencing and annotation of the polychaete Hermodice carunculata (Annelida, Amphinomidae)
Source: BMC Genomics. 2015 Jun 10;16(1):445. doi: 10.1186/s12864-015-1565-6 (PMC4462082; doi:10.1186/s12864-015-1565-6)
Supplement: Additional file 1: — Table of assigned Go terms and InterPro IDs for Open Reading Frame generated from Hermodice carunculata transcriptome. [file 12864_2015_1565_MOESM1_ESM.docx]

Transcriptome sequencing and annotation of the polychaete *Hermodice carunculata* (Annelida, Amphinomidae)

Shaadi Mehr[1,2*], Aida Verdes[3], Rob DeSalle[2], John Sparks[2,4], Vincent Pieribone[5] and David F. Gruber[2,3*]

[1]State University of New York
Biological Science Department
College at Old Westbury
Old Westbury, NY, 11568 USA

[2]American Museum of Natural History
Sackler Institute for Comparative Genomics
Central Park W at 79th St
New York, NY 10024, USA

[3]Baruch College and The Graduate Center
Department of Natural Sciences
City University of New York
New York, NY 10010, USA

[4]American Museum of Natural History
Department of Ichthyology
American Museum of Natural History
Division of Vertebrate Zoology
New York, NY 10024, USA

[5]John B. Pierce Laboratory
Cellular and Molecular Physiology
Yale University
New Haven, CT 06519, USA

Email addresses of all authors: **MehrS@oldwestbury.edu**, **averdes@gradcenter.cuny.edu**, **desalle@amnh.org**, **jsparks@amnh.org**, **vpieribo@jbpierce.org**, **David.Gruber@baruch.cuny.edu**

Correspondence: **David.Gruber@baruch.cuny.edu, MehrS@oldwestbury.edu**

Abstract

Background: The amphinomid polychaete *Hermodice carunculata* is a cosmopolitan and ecologically important omnivore in coral reef ecosystems, preying on a diverse suite of reef organisms and potentially acting as a vector for coral disease. While amphinomids are a key group for determining the root of the Annelida, their phylogenetic position has been difficult to resolve, and their publically available genomic data was scarce.

Results: We performed deep transcriptome sequencing (Illumina HiSeq) and profiling on *Hermodice carunculata* collected in the Western Atlantic Ocean. We focused this study on 58,454 predicted Open Reading Frames (ORFs) of genes longer than 200 amino acids for our homology search, and Gene Ontology (GO) terms and InterPro IDs were assigned to 32,500 of these ORFs. We used this *de novo* assembled transcriptome to recover major signaling pathways and housekeeping genes. We also identify a suite of *H. carunculata* genes related to reproduction and immune response.

Conclusions: We provide a comprehensive catalogue of annotated genes for *Hermodice carunculata* and expand the knowledge of reproduction and immune response genes in annelids, in general. Overall, this study vastly expands the available genomic data for *H. carunculata,* of which previously consisted of only 279 nucleotide sequences in NCBI. This underscores the utility of Illumina sequencing for *de novo* transcriptome assembly in non-model organisms as a cost-effective and efficient tool for gene discovery and downstream applications, such as phylogenetic analysis and gene expression profiling.

Keywords: Next-Generation Sequencing, *Hermodice carunculata,* polychaete, molecular phylogenetics, *de novo* assembly, functional annotation

Background

The amphinomid polychaete *Hermodice carunculata* (Annelida, Amphinomidae) is a cosmopolitan and ecologically important omnivore inhabiting coral reefs and other habitats throughout the Atlantic Ocean, including the Gulf of Mexico and the Caribbean Sea, as well as the Mediterranean and Red seas [1]. It is known to prey on a diverse suite of reef organisms such as zoanthids [2, 3], scleractinian corals [4–7], milleporid hydrocorals [5, 8], anemones [9] and gorgonians [5]. *Hermodice carunculata* is also a winter reservoir and spring-summer vector for the coral-bleaching pathogen *Vibrio shiloi* [10] and plays a complex and potentially ecologically important role in coral reef ecosystem health.

Amphinomidae is a well-delineated clade within aciculate polychaetes and it comprises approximately 200 described species from 25 genera [11–13]. Amphinomids are distributed worldwide and are known to inhabit intertidal, continental shelf and shallow reef communities, with a few species also recorded from the deep-sea [13]. The clade is primarily identified by a series of morphological apomorphies including nuchal organs situated on a caruncle, a ventral muscular eversible proboscis with thickened cuticle on circular lamellae, and calcareous chaetae [12, 14]. Due to the lack of knowledge regarding their morphological variability (particularly within closely related genera), previous studies based mainly on morphology have failed to clarify the evolutionary history of the group, leading to taxonomic problems. In fact, several nominal species have been regarded as conspecifics, often without evaluation of molecular data, which

might explain the common occurrence of cosmopolitan species within the clade [15]. Consequently, detailed revisions of species and even genera are needed [13], which incorporate molecular phylogenetic studies to clarify the affinities within the family [11, 16]. Additionally, amphinomids are group with unclear phylogenetic position within Annelida as different studies find different evolutionary affinities for the group [16, 17], but regarded as morphologically primitive and considered of prime interest for determining the root of the annelid Tree of Life [18]. However, the availability of genomic data in public databases for *Hermodice carunculata* and other amphinomid species is particularly scarce. Previous to this study, only 279 sequences were accessible in NCBI for *H. carunculata*..

Furthermore, the annelid *Hermodice carunculata* is a representative of the Lophotrochozoa, a clade of protostome bilaterian animals that comprises about half of the extant animal phyla, including Mollusca, the second most diverse phylum [19]. Annelids, in general, are of interest within lophotrochozoans because they are among the first coelomates [20] and polychaetes in particular, exhibit ancestral traits in body plan and embryonic development [20, 21]. Nevertheless, polychaete annelids and lophotrochozoans have been heavily underrepresented in sequencing efforts, therefore, genomic resources for this key bilaterian clade are still relatively poor compared to the other two major bilaterian clades (Ecdysozoa and Deuterostomia) [21]. A more complete representation of taxa in the genomic databases is needed to better understand animal evolution and unravel the origins of organismal diversity, especially of crucial clades such as the Lophotrochozoa [21, 22].

Here, we provide a *de novo* transcriptome assembly of *Hermodice carunculata*, a cosmopolitan Lophotrochozoan polychaete that inhabits coral reefs throughout the Atlantic Ocean. In this study we use the Illumina HiSeq platform to generate a cDNA library for *H. carunculata*. These Next-Generation Sequencing (NGS) libraries have an enormous sequencing depth and better effectiveness, producing at least 100 to 10,000 times higher throughput than classical Sanger sequencing [23]. This allows for the examination of thousands of transcripts from uncharacterized species and renders it useful for a wide range of biological applications including phylogenomics [24], regulatory gene discovery [25–28], molecular marker development [29], single nucleotide polymorphism (SNP) identification for trait adaptation [30, 31], haplotype detection [32, 33], and differential gene expression profiling [25, 32]. In this study we provide a reference set of mRNA sequences for *H. carunculata*, which will facilitate annotation of the genome and future studies of polychaete evolution, systematics and functional genomics. We specifically focused on major signaling pathways and housekeeping genes, as well as genes related to reproduction and immune response, and we provide a comprehensive list of genes related to these key processes in the annelid *H. carunculata*.

Results and Discussion

Sequencing and *de novo* assembly

Total RNA was extracted from the body-segment *H. carunculata.* The $(A)^+$ RNA was isolated, sheered to smaller fragments, and reverse transcribed to make cDNA for sequencing with Hi-Seq Illumina 1000. Four hundred million paired-end strand-unspecific reads were obtained from one lane of one plate, generating 32.4 gigabase pairs

(Gbp) of raw data that were uploaded to NCBI. Reads were checked for Phred-like quality scores above the Q30 level with FastQC [34]. We used the pipeline proposed in [35] to remove low quality reads for *de novo* assembly. HiSeq Illumina read sequences were assembled into 525,989 contigs longer than 200bp, with an N50 of 1,095 and mean length of 722.30 bp, using ABySS 1.3.1 [36], followed by Blat (with default parameters) [37] for redundancy removal. A range of 8 k-mers (21-55) were used for ABySS runs, with the parameter q = 3 to trim low-quality bases from the ends of reads for each run. The final data set was filtered for contigs longer than 200 bp. Summary statistics for each k-mer assembly, as well as for the merged and redundant-removed set of contigs is outlined in **Table 1**. Paired-end reads and assembled contigs that do not contain ambiguous bases have been deposited into NCBI and can be downloaded at the NCBI Sequence Read Archive: http://www.ncbi.nlm.nih.gov/sra/SRX194586[accn]

Assemblies at higher k-mers (e.g. 41-55) had lower mean length and N50 than assemblies at lower k-mers (21-35) (**Table 1**). This is in agreement with other summary statistics of NGS reported *de novo* assembly data [38]. The lower N50 and mean in the final merged dataset, compared with k-mer 51 and k-mer 55, is due to addition of shorter sequences from lower k-mer assemblies. As outlined in **Table 1**, the N50 has changed from 584 in k-mer 21 to 1095 bp in the merged set of contigs, indicating an improvement in the assembly contig length. Although the majority of the contig length is between 200-600 bp, we obtained 20,828 contigs, with length greater than 3,563 bp **(Fig. 1).** This result indicates that the data has a very high quality for further annotation. Lastly, the assembled sequences were deposited in Transcriptome Shotgun Assembly (TSA) at the

NCBI.

A six frame translation (ORFs) from stop to stop for each assembled contig was generated using the EMBOSS package, version: 6.4.0.0 [39]. This file contained 58,454 predicted ORFs longer than 200 AA, with the N50 of 490 AA, and mean length of 443.92 AA.

Comparative sequence similarity with other annelids

For comparative annotation, all ORFs longer than 200 AA (58,454) were initially searched against two existing annelid genomic datasets, *Capitella teleta* (http://genome.jgi-psf.org/Capca1/Capca1.home.html) and *Helobdella robusta* (http://genome.jgi-psf.org/Helro1/Helro1.home.html); and subsequently against *Paramphinome jeffreysi* and *Eurythoe complanata*, using BlastP [40] with a significant E-value of $2e^{-15}$. Similarity search showed that 23,617 (40.5%) ORFs have similarity higher than 70% against *C. teleta,* while 20,468 (35%) ORFs have similarity higher than 70% against *H. robusta* (**Fig. 2**). This indicates that the proportion of sequences with matches in the proteome of *C. teleta* is greater than the proportion of matches for *H. robusta*. This is expected, as *C. teleta* and *H. caruncula*ta are both polychaete annelids, as opposed to *H. robusta*, a leech (Clitellata). In total, 15,841 transcripts had a significant hit (70% length homology) in both datasets. Furthermore, 29,819 of these ORFs showed homology to *P. jeffreysi* and 36,033 to *E. complanata*. Of these ORFs, 23,441 were homologous to both *Paramphinome jeffreysi* and *Eurythoe complanata.* These shared sequences can be used for future genome annotation of both annelids and amphinomids,

respectively (data available upon request).

Functional annotation and characterization

One of the important aspects of mining the transcriptomic data is assigning function to individual transcripts. Functional annotation is an effective way to categorize genes into physiological classes to assist in understanding the large quantity of transcripts and for evaluating functional differences between subgroups of sequences. These data provide a tool for designing custom microarray experiments related to annotated functions [41]. Gene ontology (GO, http://www.geneontology.org) [42, 43] is an extensive scheme for this purpose. This framework covers a wide biological scope, and with its directed acyclic graph (DAG) structure, it accounts for biological dependencies. In addition, programs such as InterProScan [44, 45] provide an integrated platform for domain-based searches against databases such as PROSITE [46], PRINTS [47], Pfam [48], and SMART [49], in addition to others. Over the past few years, resources have been developed for automatic GO term and InterPro ID assignment to unknown sequences. Blast2GO [50] was utilized for functional annotation, visualization and its associated statistics.

As part of the Blast2GO pipeline, ORFs longer than 200 AA (58,454) were subjected to sequence homology search against the non-redundant protein database (NR) at NCBI, using BlastP (E 10-10, cutoff =55, GO weight=5, HSP coverage=0). Followed by mapping to collect GO terms, and assigning reliable information to each query sequence. Default values of Blast2GO annotation parameters were chosen to optimize the ratio between annotation accuracy and coverage [51]. This provided a framework for

categorizing genes into functional annotation groups, namely biological process (sets of molecular events or operations with a defined beginning and end), molecular function (the primary activities of gene product at the molecular level, such as catalysis or binding), and cellular compartment. Furthermore, InterPro IDs (protein domain IDs) were assigned to sequences by running InterProScan (part of the Blast2Go pipeline).

Out of 58,454 predicted ORFs, 55.6 % (32,500) of the data contained definitive functional annotation. These sequences were classified into three categories (GOslim): biological process, cellular component and molecular function. The summary of classification of annotation is reported at Level 2 of GO Category. In the molecular function, the clusters relating to “binding” and “catalytic activity” were enriched (21,089 and 12,443, respectively) (**Fig. 3A**). In the biological process classification, “metabolic process” with 14,272 sequences, “cellular processes” with 14,254 sequences, and “biological regulation” with 8,818 sequences were large compared to “regulation of anatomical structure size” and “cell growth” with about 200 sequences each (**Fig. 3B**). This is expected, as these data are not collected from a developmental stage with high rate of divisions. In the cellular component category, the cluster size of “cell” with 20,053 sequences and “organelle” with 11,413 sequences were highly represented compared to “microbody” or “extracellular matrix” with less than 100 sequences each (**Fig. 3C**). This pattern is very similar to a recent analysis of *Lymnae stagnalis* (pond snail) transcriptome functional annotation [26].

In terms of length distribution of annotated sequences, 70% to 90% of the sequences with

length ranging from 200 AA to 1,500 AA were functionally annotated, while 100% of the sequences with length between 1,500 AA to 3,500 AA had a GO term assigned to them (**Fig. 4).** This result indicates that longer sequences have a higher rate of annotation than shorter sequences. The annotated sequences and a table representing sequence IDs with their assigned GO terms and InterPro IDs and enzyme codes are reported (Supp. S1).

Identification of Candidate Genes and Potential Phylogenetic Markers

Signaling Pathway and Housekeeping Genes

We identified 21 homologs of housekeeping genes belonging to CAT, MAT, PFK, ATP Synthase and 4,450 homologs of signaling pathways belonging to Activin, Deltex, DPP, Fringe, Jagged, Notch, Notch2, SMAD, TGF- β; (see Supp. Table 1). Riesgo and colleagues [52], in their analysis of ten transcriptomes of newly sequenced invertebrates, found similar homologs in mollusk and annelid transcriptomes.

Immune Response Genes

We identified 172 orthologous sequences of 37 genes involved in immune response (Supp. Table 1), including caspase, interleukin, toll-like receptors, IRF genes, ficolin, antistasin and angiopoietin among others.

Reproduction Genes

We identified 46 homologous sequences to 17 genes involved in reproduction, including attractin, vasa, germ cell-less, piwi, smaug, nanos, zona pellucida, spermatogenesis-associated proteins and zonadhesin (Supp Table 1).

Potential Phylogenetic Markers

Using reciprocal BLAST searches between the *Hermodice carunculata* transcriptome and publicly available sequences, we have identified putative *H. carunculata* homologues of genes that have been previously used as phylogenetic markers in Annelida but were unavailable for *H. carunculata* and amphinomids in general, with a few exceptions. We identified 900 homologous sequences of EF-1α, 101 homologous to H3, 7 homologous to CytB, and 400 homologous to U2 snRNA. We chose the longest sequence in each category for downstream phylogenetic analysis. The alignment of each of these sequences, along with the five best hits retrieved by BLAST from the NCBI database, are available in the supplementary materials (Supp. S2-S5). Sequences were deposited in GeneBank.

Light Production Genes

A search for sequence homology in the transcriptome of *Hermodice carunculata* against 182 known bioluminescent-related proteins, such as the photoproteins Obelin, Aequorin, and other luciferases, found eight sequence transcripts with an average of 44.9% homology to the luciferase protein of the phylogenetically distant sea pansy *Renilla reniformis* (Cnidaria, Renillidae). An alignment of the *H. carunculata* putative luciferase with *Renilla* luciferase is generated (**Fig. 6**) and the corresponding cDNA sequences are included (Supp. S6).

***In silico* Quantification of the *Hermodice carunculata* Transcriptome**

In order to identify poor quality and potentially misassembled transcripts, reads were mapped back onto the non-redundant set of transcripts [53]. The number of reads corresponding to each transcript ranged from 2 to 9000 with an average of 1,644 reads, indicating a wide range of expression (Supp. S7). This indicates that very low expressed transcripts were represented in our dataset. Furthermore, we analyzed the coverage of the functionally annotated transcripts. The minimum coverage was 2 FPKM and maximum was 20,000 FPKM. Among these, 400 transcripts had a mean coverage less than 3, or gaps were removed from dataset (**Table 2**).

Conclusions

Relying on Next Generation Sequencing techniques and a thorough bioinformatics pipeline we have generated a comprehensive list of major signaling pathways, housekeeping genes, and genes related to reproduction and immune response in a representative of the Lophotrochozoa, the polychaete annelid *Hermodice carunculata*, whose phylogenetic placement within Annelida has been difficult to resolve. Major signaling pathways are highly evolutionarily conserved across Metazoa and play an important role during embryonic and adult development, regulating many fundamental cellular processes such as proliferation, stem cell maintenance, differentiation, migration or apoptosis [54]. In addition, some genes such as those involved in Notch signaling might have a role in segment formation and adult regeneration in polychaetes [55]. Housekeeping genes are required for the maintenance of essential basal cellular functions and consequently, under normal conditions, they are expressed in all cells regardless of tissue type or developmental stage [56]. They are especially interesting because they

represent the minimal set of genes required to sustain life and they can be used as comparative controls for experimental and computational studies [56], for example, to assess the suitability of transcriptome datasets for gene discovery [52]. Immune response genes are also of great concern especially among invertebrates because they represent an early model of the more highly evolved innate immune system of vertebrates [57]. Knowledge of the invertebrate immune system is based mainly in two ecdysozoan model organisms, *Drosophila melanogaster* and *Caenorabditis elegans*, and although Lophotrochozoan systems show some distinct differences [58], studies focusing on this group are very limited. Lastly, characterization of the reproductive genes of polychaetes is of interest as they exhibit an astonishing diversity of reproductive strategies, including both sexual and asexual reproduction, and range from spawning and external fertilization to brooding or viviparism, often involving marked morphological, physiological and behavioral modifications [12]. For example, some amphinomids such as *Eurythoe complanata* or *Cryptonome conclava* exhibit both sexual and asexual reproduction, the latter accomplished by architomic scissiparity: the body fragments in two or more parts which regenerate head, tail or both [13, 59].

Sex pheromones have been postulated to drive cryptic speciation in oligochaetes [60]. Within polychaetes, there are several species known to use pheromones to attract the opposite sex and to control the release of gametes, such as the scale worm *Harmothoe imbricata* [61], the rag worms *Nereis succinea* and *Platynereis dumerilii* and the lugworm *Arenicola marina* [62]. The sex pheromone attractin has been suggested by previous authors as a potential phylogenetic marker [60]. As part of our annotation

pipeline, we have identified seven sequences homologous to attractin in the transcriptome of *Hermodice carunculata*. A phylogenetic analysis was performed to evaluate the potential of the *H. carunculata* attractin protein as a reliable phylogenetic marker for polychaete systematics and evolutionary studies. Our analysis corroborates results by previous authors [60] suggesting that attractin represents an effective phylogenetic marker, recovering deep metazoan relationships (**Fig. 5**; Supp. S8) and important clades such as Bilateria, its split into Deuterostomia and Protostomia, and the subdivision of the latter in Ecdysozoa and Spiralia (Lophotrochozoa). Attractin also recovers Annelida as a monophyletic group (**Fig. 5**).

Several so-called cosmopolitan species within amphinomids have proven to comprise various cryptic species [1]. *Hermodice carunculata* has a widespread distribution and has been reported throughout the Atlantic Ocean, Caribbean, Mediterranean and Red Sea [63, 64]. Despite its widespread distribution, its representation in NCBI consisted of only 359 nucleotide sequences and only a handful of studies have examined genetic aspects of *H. carunculata*. For example, in a species delineation study, two mitochondrial genes (COI and 16S rDNA) and the internal transcribed spacer 1 (ITS1) were used to test for cryptic speciation in *H. carunculata* [1]. This analysis showed that genetic divergence is low among samples across the Atlantic Ocean, and these particular three genes do not reflect any genetic basis for the observed morphological differences (e.g., variable filament abundance) among populations. Therefore, identification of informative loci for phylogeographic application is necessary. However, a different study using COI sequences has found that *Eurythoe complanata* represents a complex of three genetically

distinct and morphologically indistinguishable lineages inhabiting the Atlantic and Pacific Oceans. Also, the deep-sea genus *Archinome* has been shown to comprise four genetically distinct lineages with no apparent morphological differences [65]. Therefore, the *de novo* assembled transcriptome presented herein for *Hermodice carunculata*, can also be used to develop additional molecular phylogenetic markers to aid forthcoming studies of species boundaries and evolutionary relationships within Amphinomidae. Furthermore, amphinomids are a morphologically plesiomorphic group of annelids, considered as a highly important taxon for reconstructing relationships at the base of the annelid tree [18]. Thus, the vast amount of molecular data provided herein can also help to elucidate the basal relationships of Annelida.

Within annelid polychaetes there are a number of bioluminescent species distributed in various families such as Acrocirridae (*Swima*), Chaetopteridae (*Chaetopterus*), Flabelligeridae (*Poeobius, Flota*), Polynoidae (*Harmothoe, Polynoe*), Syllidae (*Odontosyllis*, *Eusyllis, Pionosyllis*), Terebellidae (*Polycirrus*, *Thelepus*) and Tomopteridae (*Tomopteris*) [66]. To date, no bioluminescent protein sequence has been reported from this phylum, but we do report homologous sequences of a luciferase protein (**Fig. 6**). The fact that the putative *Hermodice carunculata* luciferase shows highest homology to the luciferase of a phylogenetically distant cnidarian (*Renilla reniformis*) can probably be attributable to the lack of publicly available luciferase sequences from more closely related organisms. The transcriptomic dataset presented herein can greatly help identify and characterize this putative photoprotein and facilitate future studies investigating the genetic and biochemical basis of light production in

annelids. In addition, we report both green and red biofluorescence in *Hermodice caruncculata*, yet the search of the genome showed no homology to any known fluorescent protein species (**Fig. 7**).

An additional recent approach in estimating more accurate intergeneric and intrageneric level relationships utilizes conserved blocks of homologous sequences shared between genomic regions of multiple species [67]. Our data provides a complementary resource for this kind of application in the future. Also, the annotation of the genomes is reliant on transcriptome data for the exon intron boundary delimitation. Our data provide a base for future genomic and ecological research on *Hermodice carunculata*, as well as a resource to understand the natural history of polychaetes and the evolution of annelids in general.

Materials and Methods

Sample collection

Research, collecting and export permits were obtained from the government of the Bahamas while working out of the Perry Institute for Marine Science on Lee Stocking Island during a December 2011 expedition. The sample was collected by scientific divers D. Gruber, J. Sparks and M. Lombardi from Norman's Pond Cay Cave, Norman's Pond Cay, Exumas, Bahamas (GPS N 23 47.181, W 076 08.428). The cave's entrance is a 2m by 8m sinkhole located just above high tide level and the cave is approximately 50 m linear and to a depth of 40 m. Divers explored the walls of the cavern zone using compact LED lights for cryptic invertebrate specimens. The *Hermodice carunculata* specimen was collected 30 m within the cave, transported back to the field station where it was frozen in liquid nitrogen less than two hours following collection.

RNA Extraction and Transcriptome Sequencing

Total RNA was extracted from dissected tail muscles. The muscle tissue was homogenized in TriZol reagent (Life Technologies, NY) and the total RNA was precipitated with isopropanol and dissolved in ddH_2O. The quality of RNA was assessed on a 2100 Bioanalyzer and with agarose gel electrophoresis. The total RNA was pooled for Library preparation. Libraries were prepared using a HiSeq RNA sample preparation kit (Illumina Inc, San Diego, CA) according to the manufacturer's instructions. One lane was multiplexed for four samples and was sequenced as 80-bp PE reads. FASTQ file generation was performed by CASAVA version 1.8.2 (Illumina).

***De novo* Assembly**

All the assemblies were performed on a server with 50 cores and 250 GB random access memory. Obtained reads were *de novo* assembled, using ABySS [36] followed by Blat version: 34x12 [37], according to the proposed pipeline for merge and redundancy removal [35] in contigs generated by ABySS. In order to recover high and low expressed transcripts, a range of k-mers (21-55) was used prior to merge with Blat.

Phylogenetic Analysis

Sequences for the sex pheromone attractin were downloaded from GenBank (accession number generation in progress) and aligned with the *Hermodice carunculata* translated sequence using MUSCLE in SEAVIEW 4.3.0 [68]. A phylogenetic analysis using amino acid sequences was conducted with RAxML ver. 7.7.1 [69] using the maximum

likelihood optimality criterion with a JTT amino acid substitution model. Support values were estimated using a rapid bootstrap algorithm with 1,000 replicates. The protozoan symbiont *Capsaspora owczarzaki* was specified as the outgroup.

Functional Annotation

Gene ontology (GO) terms and InterPro IDs were assigned to ORF sequences longer than 200 AA, using Blast2GO [50].

Availability of Supporting Data

Hermodice carunculata paired-end reads and assembled contigs can be downloaded at the NCBI Sequence Read Archive: http://www.ncbi.nlm.nih.gov/sra/SRX194586[accn]. We have also made available at LabArchives (https://mynotebook.labarchives.com/share/smehr/MjAuOHw4NTE4MS8xNi9UcmVlTm9kZS8yNzE4MjI2NjQ1fDUyLjg=): 1) a Fasta file of homologous of contigs shared between *Capitella teleta*, *Helobdella robusta* and *Hermodice carunculata*; 2) a Fasta file of homologous contigs shared between *Eurythoe complanata*, *Paramphinome jeffreysii* and *Hermodice carunculata*; and 3) the functionally annotated Open Reading Frames generated from the *Hermodice carunculata* transcriptome.

Competing Interests

The authors declare that they have no competing interests.

Authors' contributions

DFG, RD and SFPM designed the study. DFG, JS, SFPM and VAP participated in sample collection and Illumina sequencing. SFPM and DFG carried out the molecular genetic studies. SFPM and AV performed sequence alignments and phylogenetic analysis. DFG, SFPM, RD, AV and SFPM drafted the manuscript. All authors read and approved the final manuscript.

Acknowledgements

We thank Ana Riesgo for her helpful comments, Zhou Han for laboratory assistance, Mike Lombardi for assistance with sample collection assistance and the staff of the John H. Perry Caribbean Research Center for hosting our field visit to the Bahamas. This work was supported by City University of New York Collaborative Incentive Research Grant #2064, PSC-CUNY Research Award # 66474-00 44, National Geographic Society/Waitt Grant W101-10 and National Science Foundation Grant to DFG, to the Sackler Institute for Comparative Genomics and the Korein Foundation to RD and the American Museum of Natural History to JSS.

References

1. Ahrens JB, Borda E, Barroso R, Paiva PC, Campbell AM, Wolf A, Nugues MM, Rouse GW, Schulze A: **The curious case of Hermodice carunculata (Annelida: Amphinomidae): evidence for genetic homogeneity throughout the Atlantic Ocean and adjacent basins**. *Mol Ecol* 2013, **22**:2280–2291.

2. Sebens KP: **Intertidal distribution of zoanthids on the Caribbean coast of Panama: effects of predation and desiccation**. *Bull Mar Sci* 1982, **32**:316–335.

3. Karlson RH: **Disturbance and monopolization of a spatial resource by Zoanthus sociatus (Coelenterata, Anthozoa)**. *Bull Mar Sci* 1983, **33**:118–131.

4. Ott B, Lewis JB: **The importance of the gastropod Coralliophila abbreviata (Lamarck) and the polychaete Hermodice carunculata (Pallas) as coral reef predators**. *Can J Zool* 1972, **50**:1651–1656.

5. Rylaarsdam KW: **Life histories and abundance patterns of colonial corals on Jamaican reefs**. *Mar Ecol Prog Ser Oldend* 1983, **13**:249–260.

6. Wolf AT, Nugues MM: **Predation on coral settlers by the corallivorous fireworm Hermodice carunculata**. *Coral Reefs* 2012.

7. Marsden JR: **The digestive tract of Hermodice carunculata (Pallas). Polychaeta: Amphinomidae**. *Can J Zool* 1963, **41**:165–184.

8. Lewis J, Crooks R: **Foraging cycles of the amphinomid polychaete *Hermodice caruncluata* preying on the calcereous hydrozoan *Millepora complenata***. *Bull Mar Sci* 1996, **58**:853–856.

9. Fauchald K, Jumars PA: **The diet of worms: a study of polychaete feeding guilds**. 1979.

10. Sussman M, Loya Y, Fine M, Rosenberg E: **The marine fireworm Hermodice carunculata is a winter reservoir and spring-summer vector for the coral-bleaching pathogen Vibrio shiloi**. *Environ Microbiol* 2003, **5**:250–255.

11. Wiklund H, Nygren A, Pleijel F, Sundberg P: **The phylogenetic relationships between Amphinomidae, Archinomidae and Euphrosinidae (Amphinomida: Aciculata: Polychaeta), inferred from molecular data**. *J Mar Biol Assoc UK* 2008, **88**:509–513.

12. Rouse G, Pleijel F: *Polychaetes*. Oxford University Press; 2001.

13. Borda E, Kudenov JD, Bienhold C, Rouse GW: **Towards a revised Amphinomidae (Annelida, Amphinomida): description and affinities of a new genus and species from the Nile Deep-sea Fan, Mediterranean Sea**. *Zool Scr* 2012, **41**:307–325.

14. Rouse GW, Fauchald K: **Cladistics and polychaetes**. *Zool Scr* 1997, **26**:139–204.

15. Yáñez-Rivera B, Salazar-Vallejo SI: **Revision of Hermodice Kinberg, 1857 (Polychaeta: Amphinomidae)**. *Sci Mar* 2011, **75**:251–262.

16. Weigert A, Helm C, Meyer M, Nickel B, Arendt D, Hausdorf B, Santos SR, Halanych KM, Purschke G, Bleidorn C, Struck TH: **Illuminating the Base of the Annelid Tree Using Transcriptomics**. *Mol Biol Evol* 2014, **31**:1391–1401.

17. Struck TH, Paul C, Hill N, Hartmann S, Hosel C, Kube M, Lieb B, Meyer A, Tiedemann R, Purschke G, Bleidorn C: **Phylogenomic analyses unravel annelid evolution**. *Nature* 2011, **471**:95–U113.

18. Colgan DJ, Hutchings PA, Beacham E: **Multi-gene analyses of the phylogenetic relationships among the Mollusca, Annelida, and Arthropoda**. *Zool Sci* 2008, **47**:338–351.

19. Giribet G: **Assembling the lophotrochozoan (=spiralian) tree of life**. *Philos Trans R Soc L B Biol Sci* 2008, **363**:1513–1522.

20. Salzet M, Tasiemski A, Cooper E: **Innate immunity in lophotrochozoans: the annelids**. *Curr Pharm Des* 2006, **12**:3043–3050.

21. Gagniere N, Jollivet D, Boutet I, Brelivet Y, Busso D, Da Silva C, Gaill F, Higuet D, Hourdez S, Knoops B, Lallier F, Leize-Wagner E, Mary J, Moras D, Perrodou E, Rees J-F, Segurens B, Shillito B, Tanguy A, Thierry J-C, Weissenbach J, Wincker P, Zal F, Poch O, Lecompte O: **Insights into metazoan evolution from alvinella pompejana cDNAs**. *BMC Genomics* 2010, **11**:634.

22. Takahashi T, McDougall C, Troscianko J, Chen W-C, Jayaraman-Nagarajan A, Shimeld S, Ferrier D: **An EST screen from the annelid Pomatoceros lamarckii reveals patterns of gene loss and gain in animals**. *BMC Evol Biol* 2009, **9**:240.

23. Metzker ML: **Sequencing technologies—the next generation**. *Nat Rev Genet* 2009, **11**:31–46.

24. Dunn CW, Hejnol A, Matus DQ, Pang K, Browne WE, Smith SA, Seaver E, Rouse GW, Obst M, Edgecombe GD, Sorensen M V, Haddock SH, Schmidt-Rhaesa A, Okusu A, Kristensen RM, Wheeler WC, Martindale MQ, Giribet G: **Broad phylogenomic sampling improves resolution of the animal tree of life**. *Nature* 2008, **452**:745–749.

25. Feng C, Chen M, Xu CJ, Bai L, Yin XR, Li X, Allan AC, Ferguson IB, Chen KS: **Transcriptomic analysis of Chinese bayberry (Myrica rubra) fruit development and ripening using RNA-Seq**. *BMC Genomics* 2012, **13**:19.

26. Sadamoto H, Takahashi H, Okada T, Kenmoku H, Toyota M, Asakawa Y: **De novo sequencing and transcriptome analysis of the central nervous system of mollusc Lymnaea stagnalis by deep RNA sequencing.** *PLoS One* 2012, **7**:e42546.

27. Shi CY, Yang H, Wei CL, Yu O, Zhang ZZ, Jiang CJ, Sun J, Li YY, Chen Q, Xia T, Wan XC: **Deep sequencing of the Camellia sinensis transcriptome revealed candidate genes for major metabolic pathways of tea-specific compounds**. *BMC Genomics* 2011, **12**:131.

28. Crawford JE, Guelbeogo WM, Sanou A, Traore A, Vernick KD, Sagnon N, Lazzaro BP: **De novo transcriptome sequencing in Anopheles funestus using Illumina RNA-seq technology**. *PLoS One* 2010, **5**:e14202.

29. Franchini P, Van der Merwe M, Roodt-Wilding R: **Transcriptome characterization of the South African abalone Haliotis midae using sequencing-by-synthesis**. *BMC Res Notes* 2011, **4**:59.

30. Salem M, Vallejo RL, Leeds TD, Palti Y, Liu S, Sabbagh A, Rexroad 3rd CE, Yao J: **RNA-Seq identifies SNP markers for growth traits in rainbow trout**. *PLoS One* 2012, **7**:e36264.

31. Renaut S, Nolte AW, Rogers SM, Derome N, Bernatchez L: **SNP signatures of selection on standing genetic variation and their association with adaptive phenotypes along gradients of ecological speciation in lake whitefish species pairs (Coregonus spp.)**. *Mol Ecol* 2011, **20**:545–559.

32. Yang SS, Tu ZJ, Cheung F, Xu WW, Lamb JFS, Jung H-JG, Vance CP, Gronwald JW: **Using RNA-Seq for gene identification, polymorphism detection and transcript profiling in two alfalfa genotypes with divergent cell wall composition in stems**. *BMC Genomics* 2011, **12**:199.

33. Canovas A, Rincon G, Islas-Trejo A, Wickramasinghe S, Medrano JF: **SNP discovery in the bovine milk transcriptome using RNA-Seq technology**. *Mamm Genome* 2010, **21**:592–598.

34. Andrews S: **A quality control tool for high throughput sequence data.** 2010.

35. Swaminathan K, Chae WB, Mitros T, Varala K, Xie L, Barling A, Glowacka K, Hall M, Jezowski S, Ming R: **A framework genetic map for Miscanthus sinensis from RNAseq-based markers shows recent tetraploidy**. *BMC Genomics* 2012, **13**:142.

36. Simpson JT, Wong K, Jackman SD, Schein JE, Jones SJM, Birol I: **ABySS: a parallel assembler for short read sequence data.** *Genome Res* 2009, **19**:1117–1123.

37. Kent WJ: **BLAT--the BLAST-like alignment tool**. *Genome Res* 2002, **12**:656–664.

38. Surget-Groba Y, Montoya-Burgos JI: **Optimization of de novo transcriptome assembly from next-generation sequencing data**. *Genome Res* 2010, **20**:1432–1440.

39. Rice P, Longden I, Bleasby A: **EMBOSS: the European Molecular Biology Open Software Suite**. *Trends Genet* 2000, **16**:276–277.

40. Altschul S: **Gapped BLAST and PSI-BLAST: a new generation of protein database search programs**. *Nucleic Acids Res* 1997, **25**:3389–3402.

41. Nagaraj SH, Gasser RB, Ranganathan S: **A hitchhiker's guide to expressed sequence tag (EST) analysis**. *Brief Bioinform* 2007, **8**:6–21.

42. Ashburner M, Ball CA, Blake JA, Botstein D, Butler H, Cherry JM, Davis AP, Dolinski K, Dwight SS, Eppig JT: **Gene Ontology: tool for the unification of biology**. *Nat Genet* 2000, **25**:25–29.

43. Harris MA, Clark J, Ireland A, Lomax J, Ashburner M, Foulger R, Eilbeck K, Lewis S, Marshall B, Mungall C, Richter J, Rubin GM, Blake JA, Bult C, Dolan M, Drabkin H, Eppig JT, Hill DP, Ni L, Ringwald M, Balakrishnan R, Cherry JM, Christie KR, Costanzo MC, Dwight SS, Engel S, Fisk DG, Hirschman JE, Hong EL, Nash RS, et al.: **The Gene Ontology (GO) database and informatics resource**. *Nucleic Acids Res* 2004, **32**(Database issue):D258–61.

44. Zdobnov EM, Apweiler R: **InterProScan–an integration platform for the signature-recognition methods in InterPro**. *Bioinformatics* 2001, **17**:847–848.

45. Mulder NJ, Apweiler R: **The InterPro database and tools for protein domain analysis**. *Curr Protoc Bioinforma* 2008, **Chapter 2**:Unit 2 7.

46. Hofmann K, Bucher P, Falquet L, Bairoch A: **The PROSITE database, its status in 1999**. *Nucleic Acids Res* 1999, **27**:215–219.

47. Attwood TK, Croning MDR, Flower DR, Lewis AP, Mabey JE, Scordis P, Selley JN, Wright W: **PRINTS-S: the database formerly known as PRINTS**. *Nucleic Acids Res* 2000, **28**:225–227.

48. Bateman A, Coin L, Durbin R, Finn RD, Hollich V, Griffiths-Jones S, Khanna A, Marshall M, Moxon S, Sonnhammer ELL: **The Pfam protein families database**. *Nucleic Acids Res* 2004, **32**(suppl 1):D138–D141.

49. Letunic I, Copley RR, Schmidt S, Ciccarelli FD, Doerks T, Schultz J, Ponting CP, Bork P: **SMART 4.0: towards genomic data integration**. *Nucleic Acids Res* 2004, **32**(suppl 1):D142–D144.

50. Conesa A, Götz S, García-Gómez JM, Terol J, Talón M, Robles M: **Blast2GO: a universal tool for annotation, visualization and analysis in functional genomics research**. *Bioinformatics* 2005, **21**:3674–3676.

51. Conesa A, Götz S: **Blast2GO: A comprehensive suite for functional analysis in plant genomics**. *Int J Plant Genomics* 2008, **619832. doi:10.1155/2008/619832**.

52. Riesgo A, Andrade SC, Sharma PP, Novo M, Perez-Porro AR, Vahtera V, Gonzalez VL, Kawauchi GY, Giribet G: **Comparative description of ten transcriptomes of newly sequenced invertebrates and efficiency estimation of genomic sampling in non-model taxa**. *Front Zool* 2012, **9**:33.

53. Mehr SF, DeSalle R, Kao H-T, Narechania A, Han Z, Tchernov D, Pieribone V, Gruber DF: **Transcriptome deep-sequencing and clustering of expressed isoforms from Favia corals**. *BMC Genomics* 2013, **14**:546.

54. Borggrefe T, Oswald F: **The Notch signaling pathway: transcriptional regulation at Notch target genes**. *Cell Mol Life Sci* 2009, **66**:1631–1646.

55. Thamm K, Seaver EC: **Notch signaling during larval and juvenile development in the polychaete annelid Capitella sp. I**. *Dev Biol* 2008, **320**:304–318.

56. Eisenberg E, Levanon EY: **Human housekeeping genes, revisited**. *Trends Genet* 2013, **29**:569–574.

57. Cooper EL: **Comparative immunology**. *Integr Comp Biol* 2003, **43**:278–280.

58. Nyholm S V, Graf J: **Knowing your friends: invertebrate innate immunity fosters beneficial bacterial symbioses**. *Nat Rev Microbiol* 2012, **10**:815–827.

59. Kudenov JD: **The reproductive biology of Eurythoe complanata (Pallas, 1766), (Polychaeta: Amphinomidae).** University of Arizona; 1974.

60. Novo M, Riesgo A, Fernández-Guerra A, Giribet G: **Pheromone evolution, reproductive genes, and comparative transcriptomics in Mediterranean earthworms (Annelida, Oligochaeta, Hormogastridae)**. *Mol Biol Evol* 2013, **30**:1614–1629.

61. Watson GJ, Langford FM, Gaudron SM, Bentley MG: **Factors influencing spawning and pairing in the scale worm Harmothoe imbricata (Annelida: Polychaeta)**. *Biol Bull* 2000, **199**:50–58.

62. Zeeck E, Hardege J, Bartels-Hardege H: **Pla tyn ereis d urn erilii**. *Mar Ecol Prog Ser* 1990, **67**:183–188.

63. Costello MJ, Bouchet P, Boxshall G, Fauchald K, Gordon D, Hoeksema BW, Poore GC, van Soest RW, Stohr S, Walter TC, Vanhoorne B, Decock W, Appeltans W: **Global Coordination and Standardisation in Marine Biodiversity through the World Register of Marine Species (WoRMS) and Related Databases**. *PLoS One* 2013, **8**:e51629.

64. Barroso R, Paiva PC: **Amphinomidae (Annelida: Polychaeta) from Rocas Atoll, Northeastern Brazil**. *Arq Mus Nac* 2007, **65**:357–362.

65. Borda E, Kudenov JD, Chevaldonne P, Blake JA, Desbruyeres D, Fabri MC, Hourdez S, Pleijel F, Shank TM, Wilson NG, Schulze A, Rouse GW: **Cryptic species of Archinome (Annelida: Amphinomida) from vents and seeps**. *Proceedings of the Royal Society of London B.* 2013, **280**:20131876.

66. Shimomura O: *Bioluminescence: Chemical Principles and Methods*. World Scientific Publishing Company; 2012.

67. Castresana J: **Selection of conserved blocks from multiple alignments for their use in phylogenetic analysis**. *Mol Biol Evol* 2000, **17**:540–552.

68. Gouy M, Guindon S, Gascuel O: **SeaView version 4: a multiplatform graphical user interface for sequence alignment and phylogenetic tree building**. *Mol Biol Evol* 2010, **27**:221–224.

69. Stamatakis A: **RAxML-VI-HPC: maximum likelihood-based phylogenetic analyses with thousands of taxa and mixed models.** *Bioinformatics* 2006, **22**:2688–2690.

Figure legends

Figure 1: Assembled contig length distribution. Each number on top of each bar represents number of assembled contigs per length category.

Figure 2: Venn diagram distribution of similarity search results for *Hermodice caruncula*ta. Based on 58,454 predicted Open Reading Frames (ORFs) of genes longer than 200 amino acids. The number of unique sequence-based annotation is the best sum of unique BlastP hits (E-value of $2e^{-15}$) from *Capitella teleta* and *Helobdella robusta* proteome, respectively.

Figure 3A: Functional annotation of *Hermodice carunculata* transcripts -- molecular function. The 55 most frequent GOslim terms based on molecular function.

Figure 3B: Functional annotation of *Hermodice carunculata* transcripts -- biological processes. The 55 most frequent GOslim terms based on biological processes.

Figure 3C: Functional annotation of *Hermodice carunculata* transcripts -- cellular. The 55 most frequent GOslim terms based on cellular components.

Figure 4: Percentage of functionally annotated transcripts relative to their length.

Figure 5: Maximum likelihood tree of 21 Attractin proteins and one newly identified attractin sequence from *Hermodice carunculata*. The newly identified attractin is colored red.

Figure 6: Overlapping region of amino acid sequence alignment of homologous proteins sequences to luciferase from the sea pansy, *Renilla* sp.

Figure 7: Fluorescent macro image of *Hermodice carunculata* using 450-500 nm excitation and 514nm LP emission (A); white light image (B); and fluorescent macro comparison (using 450-500nm excitation and 514nmLP emission) (C); confocal images (D-G) obtained with a Olympus Fluoview FV1000 (Olympus, Japan) confocal laser scanning microscope using an Olympus LUMFL 60×/1.10 W objective (excitation 488 nm wavelength Ar-laser was used), illustrating distrubution of green and red fluorescence; (H) Emission spectra using an Ocean Optics USB2000+ miniature spectrometer (Dunedin, FL) equipped with a hand-held fiber optic probe (Ocean Optics ZFQ-12135).

Tables

Table 1

Summary Statistics for individual and merged assemblies

Assembly	Number of transcripts> 200bp	N50 bp	Mean length bp	Max length bp	Total number of bp
K-mer 21	143,191	584	505.54	7,342	72,390,913
K-mer 25	160,583	771	605.87	13,382	97,292,569
K-mer 29	188,890	631	523.05	8,878	98,798,757
K-mer 35	225,756	689	551.61	11,724	124,529,844
K-mer 41	179,143	891	633.86	18,825	113,522,250
K-mer 45	171,154	983	667.66	24,711	114,273,429

K-mer 51	156,387	1,096	713.03	17,800	111,509,378
K-mer 55	144,565	1,160	740.32	14,922	107,023,822
Final	525,989	1,095	722.3	24,711	379,922,870

**Generated ORFs from Assembly**	**Number of ORFs >200 AA**	**N50 AA**	**Mean length AA**	**Max length AA**	**Total number of AA**
ORFs>200AA	58,454	490	443.92	8,167	25,948,636

For each k-mer, data from AbySS is shown. The final assmbly is the result of merging the AbySS k-mer assemblies using BLAT to remove the redundancies. Predicted ORF's longer than 200AA's from this final contig set were used for annotation. K-mer =required length of overlap match between two reads in AbySS; N50=length weigthed median contig length; bp=base pair; ORF= Open Reading Frame

Table 2

Summary statistics of read counts and coverage

Total number of reads	426,555,924
Number of read used reads for assembly	141,684,860 (33.22%)
Number of unused reads	28,4871,064 (66.78%)
Number of non-redundant transcripts (>200 bp)	525,989
Number of non-redundant trasncripts with back-aligned reads (>200 bp)	525,939
Number of transcripts with coverage fpkm >1	176,412
Number of transcripts with coverage fpkm >5	49,690
Average coverage for contigs from filtered dataset 2 (fpmk)	15.279
Average number of reads mapped per contig (with coverage fpkm >5)	1644

bp = base pair; fpkm = paired-reads per kilobase per million; contig = contiguous overlapping sequence read from assembly

Supplimentary Data

S1: Table of assigned Go terms and InterPro IDs for Open Reading Frame generated from *Hermodice carunculata* transcriptome

S2: Fasta file of annotated EF-α expressed isoforms

S3: Fasta file of annotated Histon expressed isoforms

S4: Fasta file of annotated Cytochrome B expressed isoforms

S5: Fasta file of annotated U2 snRNA expressed isoforms

S6: Fasta file of annotated luciferases expressed isoforms

S7: RPKM of the assembled contigs

S8: Multiple Sequence Alignment of annotated attractin protein from *Hermodice carunculata* along with 21 other attractin sequences from other species.

S9: Supplementary Table 1: List of *Hermodice carunculata* reproduction and immune response genes. The specific gene, number of found contigs and the contig identification tag are included.

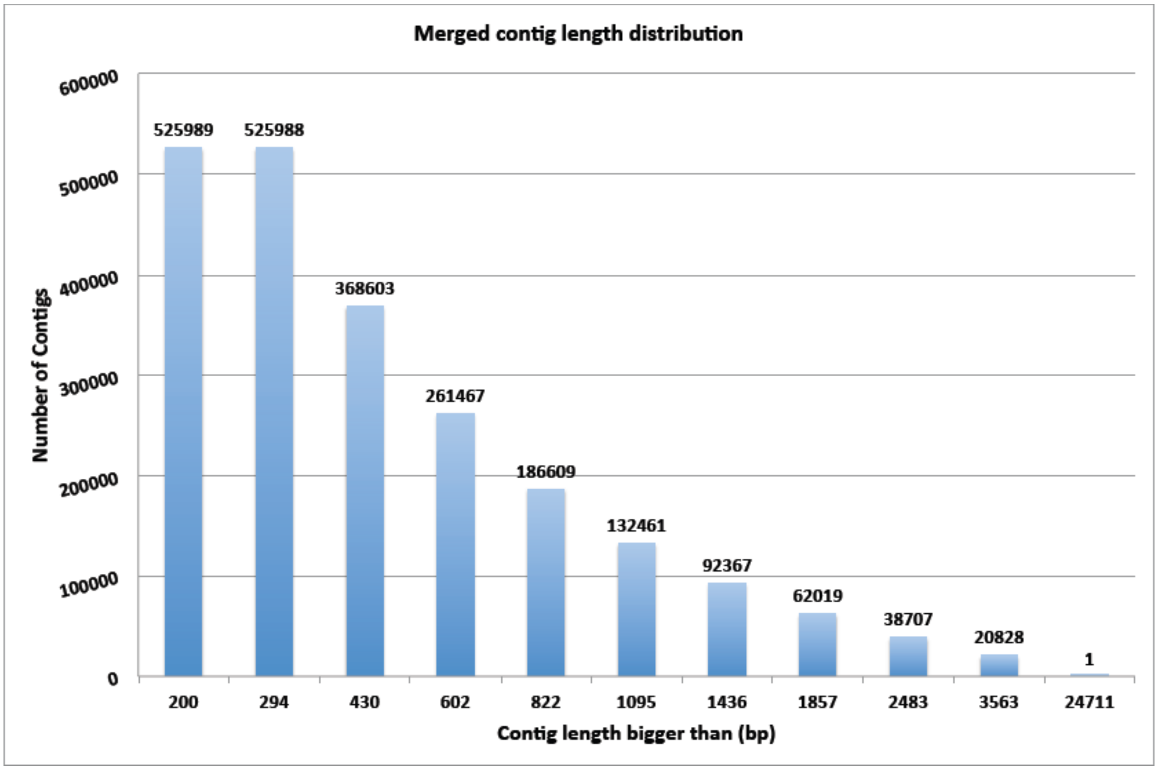
Merged contig length distribution
Number of Contigs
600000
500000
400000
300000
200000
100000
0
525989
525988
368603
261467
186609
132461
92367
62019
38707
20828
1
200
294
430
602
822
1095
1436
1857
2483
3563
24711
Contig length bigger than (bp)

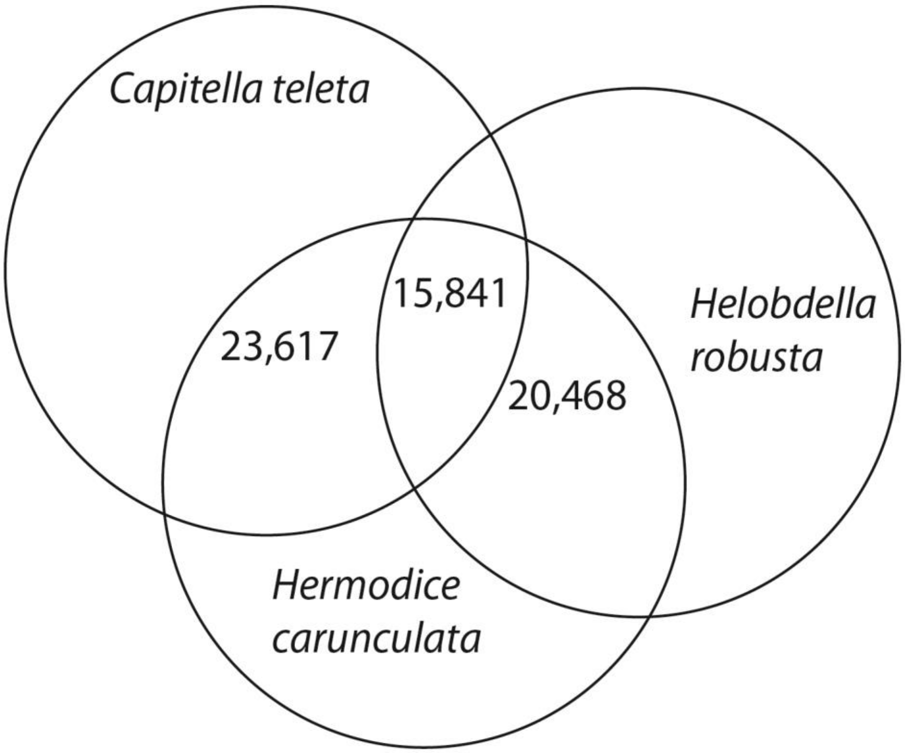
Capitella teleta
Helobdella robusta
Hermodice caruncalata
15,841
23,617
20,468

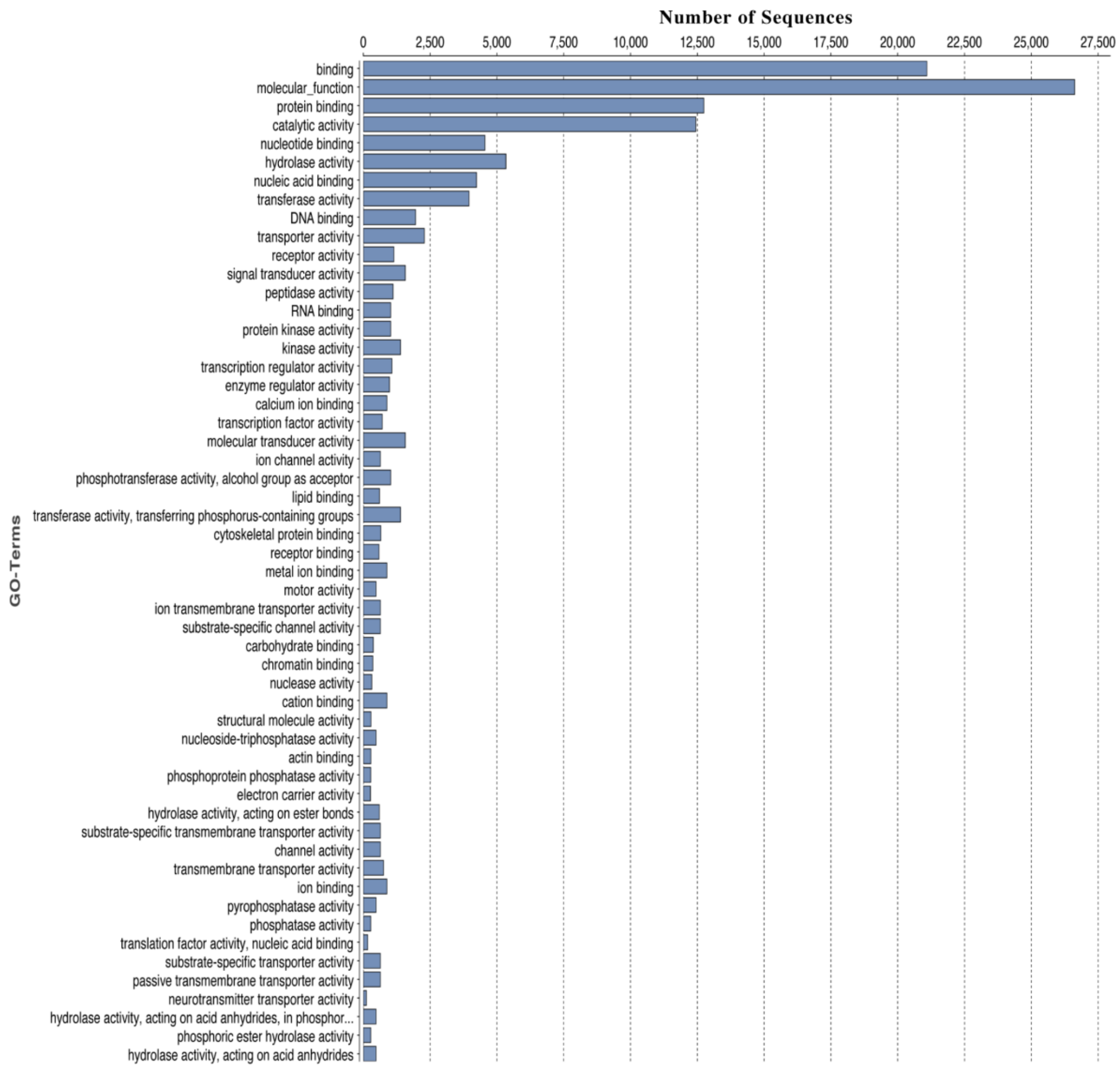
Number of Sequences
0
2,500
5,000
7,500
10,000
12,500
15,000
17,500
20,000
22,500
25,000
27,500
GO-Terms
binding
molecular_function
protein binding
catalytic activity
nucleotide binding
hydrolase activity
nucleic acid binding
transferase activity
DNA binding
transporter activity
receptor activity
signal transducer activity
peptidase activity
RNA binding
protein kinase activity
kinase activity
transcription regulator activity
enzyme regulator activity
calcium ion binding
transcription factor activity
molecular transducer activity
ion channel activity
phosphotransferase activity, alcohol group as acceptor
lipid binding
transferase activity, transferring phosphorus-containing groups
cytoskeletal protein binding
receptor binding
metal ion binding
motor activity
ion transmembrane transporter activity
substrate-specific channel activity
carbohydrate binding
chromatin binding
nuclease activity
cation binding
structural molecule activity
nucleoside-triphosphatase activity
actin binding
phosphoprotein phosphatase activity
electron carrier activity
hydrolase activity, acting on ester bonds
substrate-specific transmembrane transporter activity
channel activity
transmembrane transporter activity
ion binding
pyrophosphatase activity
phosphatase activity
translation factor activity, nucleic acid binding
substrate-specific transporter activity
passive transmembrane transporter activity
neurotransmitter transporter activity
hydrolase activity, acting on acid anhydrides, in phosphor...
phosphoric ester hydrolase activity
hydrolase activity, acting on acid anhydrides

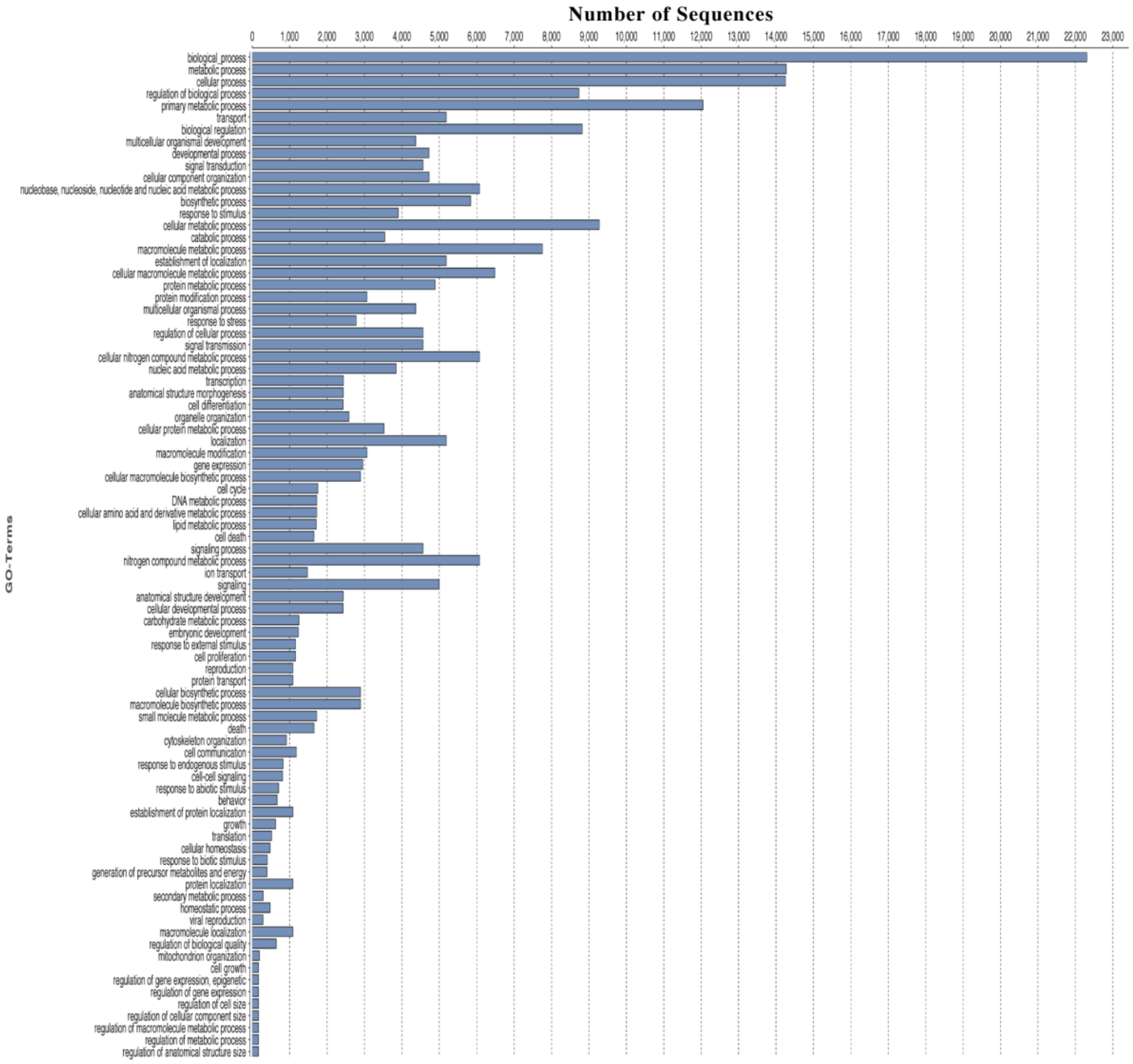
Number of Sequences
0
1,000
2,000
3,000
4,000
5,000
6,000
7,000
8,000
9,000
10,000
11,000
12,000
13,000
14,000
15,000
16,000
17,000
18,000
19,000
20,000
21,000
22,000
23,000
GO-Terms
biological_process
metabolic process
cellular process
regulation of biological process
primary metabolic process
transport
biological regulation
multicellular organismal development
developmental process
signal transduction
cellular component organization
nucleobase, nucleoside, nucleotide and nucleic acid metabolic process
biosynthetic process
response to stimulus
cellular metabolic process
catabolic process
macromolecule metabolic process
establishment of localization
cellular macromolecule metabolic process
protein metabolic process
protein modification process
multicellular organismal process
response to stress
regulation of cellular process
signal transmission
cellular nitrogen compound metabolic process
nucleic acid metabolic process
transcription
anatomical structure morphogenesis
cell differentiation
organelle organization
cellular protein metabolic process
localization
macromolecule modification
gene expression
cellular macromolecule biosynthetic process
cell cycle
DNA metabolic process
cellular amino acid and derivative metabolic process
lipid metabolic process
cell death
signaling process
nitrogen compound metabolic process
ion transport
signaling
anatomical structure development
cellular developmental process
carbohydrate metabolic process
embryonic development
response to external stimulus
cell proliferation
reproduction
protein transport
cellular biosynthetic process
macromolecule biosynthetic process
small molecule metabolic process
death
cytoskeleton organization
cell communication
response to endogenous stimulus
cell-cell signaling
response to abiotic stimulus
behavior
establishment of protein localization
growth
translation
cellular homeostasis
response to biotic stimulus
generation of precursor metabolites and energy
protein localization
secondary metabolic process
homeostatic process
viral reproduction
macromolecule localization
regulation of biological quality
mitochondrion organization
cell growth
regulation of gene expression, epigenetic
regulation of gene expression
regulation of cell size
regulation of cellular component size
regulation of macromolecule metabolic process
regulation of metabolic process
regulation of anatomical structure size

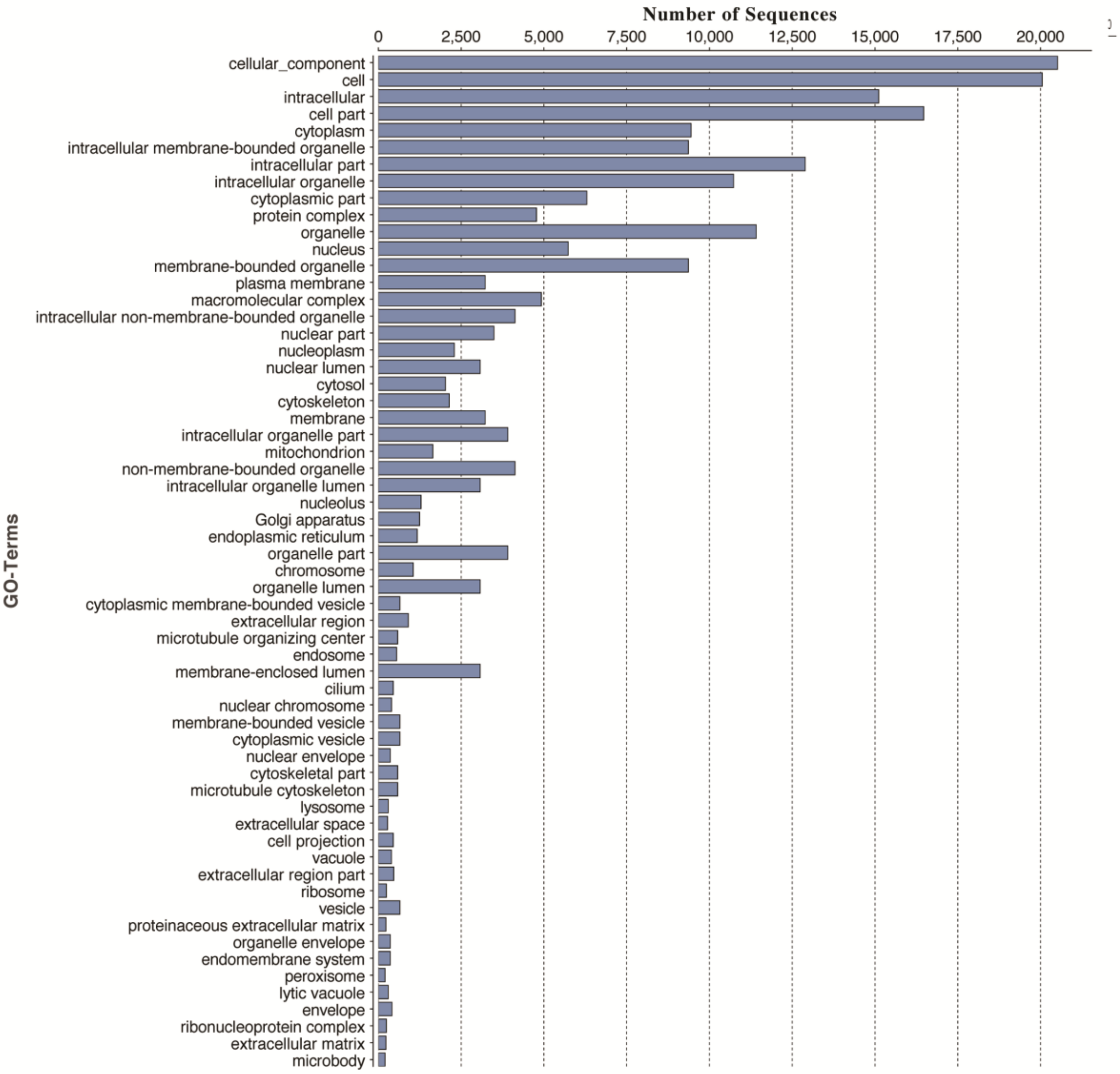
Number of Sequences
0
2,500
5,000
7,500
10,000
12,500
15,000
17,500
20,000
GO-Terms
cellular_component
cell
intracellular
cell part
cytoplasm
intracellular membrane-bounded organelle
intracellular part
intracellular organelle
cytoplasmic part
protein complex
organelle
nucleus
membrane-bounded organelle
plasma membrane
macromolecular complex
intracellular non-membrane-bounded organelle
nuclear part
nucleoplasm
nuclear lumen
cytosol
cytoskeleton
membrane
intracellular organelle part
mitochondrion
non-membrane-bounded organelle
intracellular organelle lumen
nucleolus
Golgi apparatus
endoplasmic reticulum
organelle part
chromosome
organelle lumen
cytoplasmic membrane-bounded vesicle
extracellular region
microtubule organizing center
endosome
membrane-enclosed lumen
cilium
nuclear chromosome
membrane-bounded vesicle
cytoplasmic vesicle
nuclear envelope
cytoskeletal part
microtubule cytoskeleton
lysosome
extracellular space
cell projection
vacuole
extracellular region part
ribosome
vesicle
proteinaceous extracellular matrix
organelle envelope
endomembrane system
peroxisome
lytic vacuole
envelope
ribonucleoprotein complex
extracellular matrix
microbody

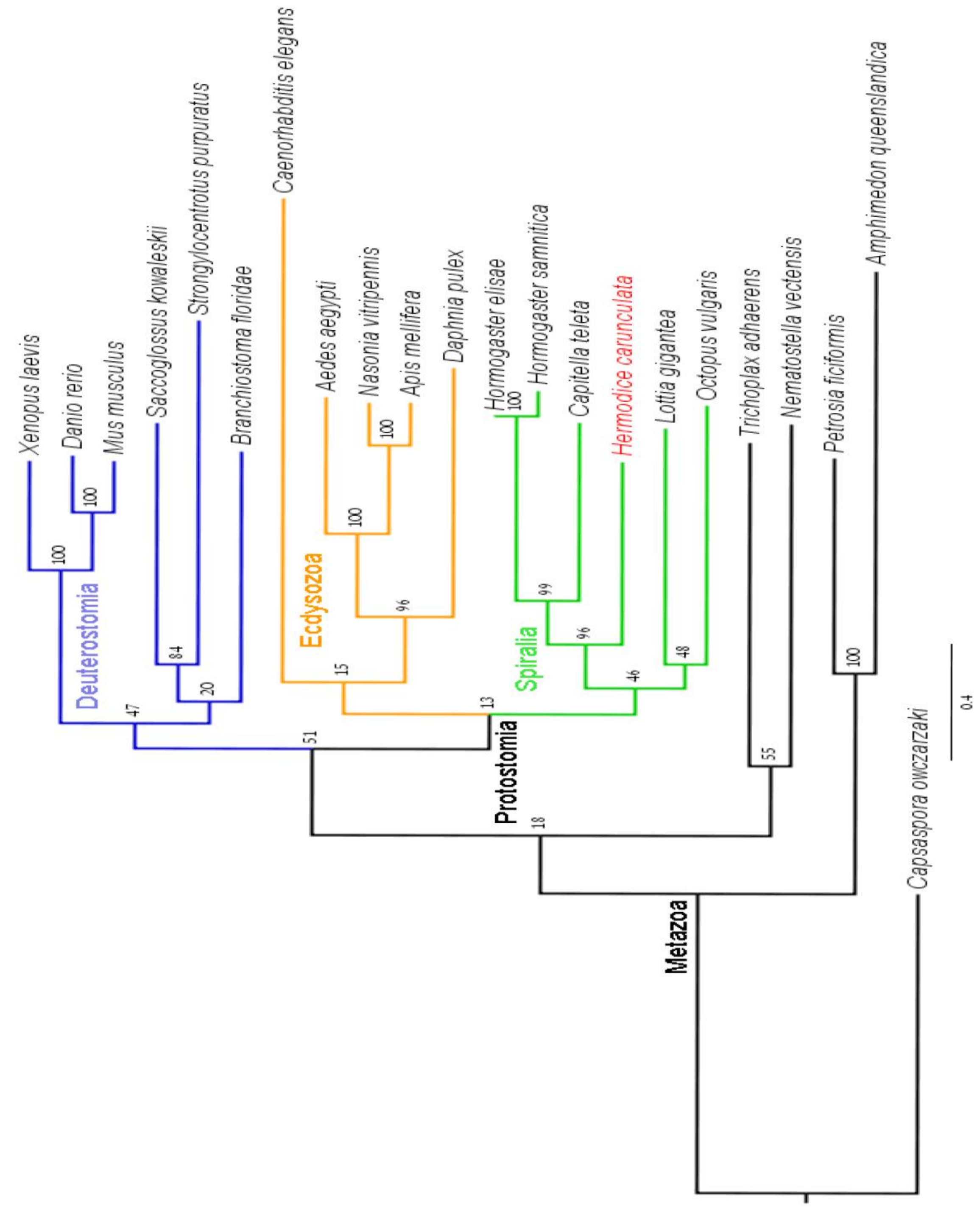

Xenopus laevis
Danio rerio
Mus musculus
Saccoglossus kowaleskii
Strongylocentrotus purpuratus
Branchiostoma floridae
Caenorhabditis elegans
Aedes aegypti
Nasonia vitripennis
Apis mellifera
Daphnia pulex
Hormogaster elisae
Hormogaster samnitica
Capitella teleta
Hermodice carunculata
Lottia gigantea
Octopus vulgaris
Trichoplax adhaerens
Nematostella vectensis
Petrosia ficiformis
Amphimedon queenslandica
Capsaspora owczarzaki
Deuterostomia
Ecdysozoa
Spiralia
Protostomia
Metazoa
0.4

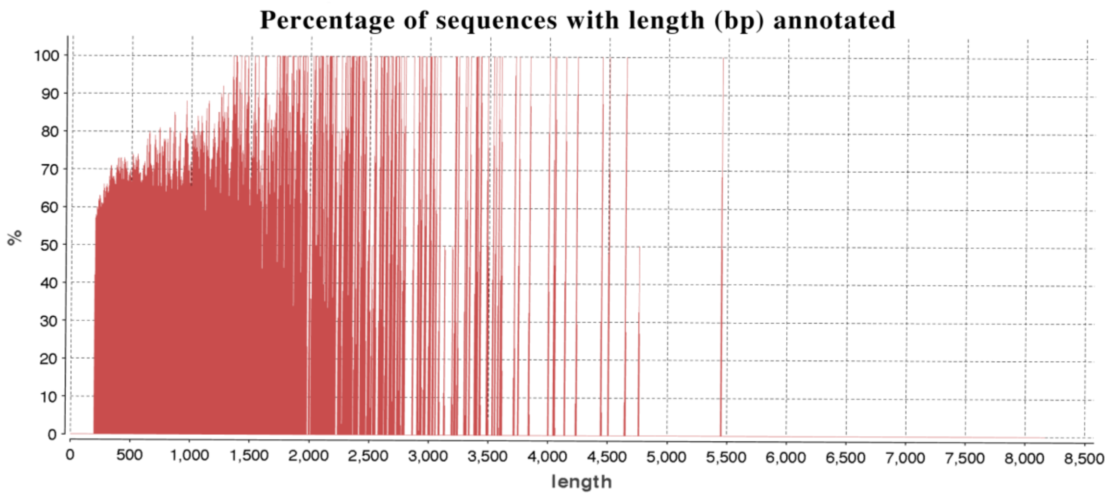
Percentage of sequences with length (bp) annotated
%
100
90
80
70
60
50
40
30
20
10
0
0
500
1,000
1,500
2,000
2,500
3,000
3,500
4,000
4,500
5,000
5,500
6,000
6,500
7,000
7,500
8,000
8,500
length

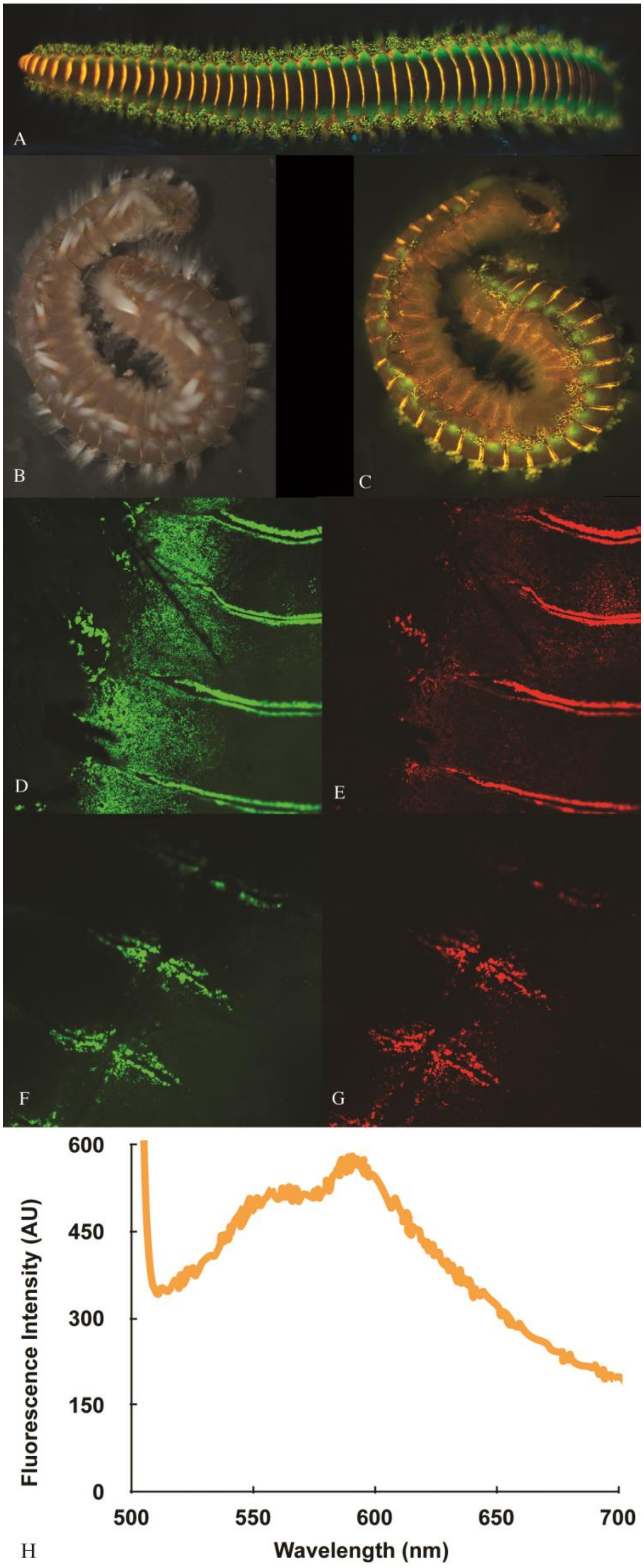

A
B
C
D
E
F
G
Fluorescence Intensity (AU)
0
150
300
450
600
500
550
600
650
700
Wavelength (nm)
H

```
Renilla_Luciferase      MTSKVYDPEQRKRMITGPQWWARCKQMNVLDSFINYYDSEKHAENAVIFLHGNAASSYLW
K55ctg4472189_6         ------------------------------------------GDNVVIFLHGNPTAAYLW
K51ctg5345809_19        ------------------------------------------GDNVVIFLHGNPTAAYLW
K45ctg7135339_19        -----------------------------------------------IFLHGNPTAAYLW
K45ctg7152679_7         -----------------------------------------------IFLHGNPTAAYLW
K51ctg5324073_5         ------------------------------------------------------------
K55ctg4446157_3         ------------------------------------------------------------
K35ctg13194577_2        ------------------------------------------------------------
K45ctg7170306_22        ------------------------------------------------------------

Renilla_Luciferase      RHVVPHIEPVARCIIPDLIGMGKSGKSGNGSYRLLDHYKYLTAWFELLNLPKKIIFVGHD
K55ctg4472189_6         RNIIPHVQPTARCLAPDLIGMGHSAKLPSHNYRFADHYRYLSAWIEKMNLPAKVSFVIHD
K51ctg5345809_19        RNIIPHVQPTARCLAPDLIGMGHSAKLPSHNYRFADHYRYLSAWIEKMNLPSKVSLVIHD
K45ctg7135339_19        RNIIPHVQPTARCLAPDLIGMGHSAKLPSHNYRFADHYRYLSAWIEKMNLPSKVSLVIHD
K45ctg7152679_7         RNIIPHVQPIARCLAPDLIGMGHSAKLPSHNYRFADHYRYLSAWIEKMNLPAKVSFVIHD
K51ctg5324073_5         RNIIPHVQPIARCLAPDLIGMGHSAKLPSHNYRFADHYRYLSAWIEKMNLPAKVSFVIHD
K35ctg13194577_2        ------------------------------------------------------------
K45ctg7170306_22        ------------------------------------------------------------

Renilla_Luciferase      WGACLAFHYSYEHQDKIKAIVHAESVVDVIESWDEWPDIEEDIALIK-SEEGEKMVLENN
K55ctg4472189_6         WGSGLGFHWSNEHRDRVQALIHMESLASCIPSWDLFPEVASNVFQALRSDAGEEMVLKKN
K51ctg5345809_19        WGSGLGFHWSNEHRDRVQALVHMESVVRPVLSWDRFPEVARNIFQALRSDAGEEIVLQKN
K45ctg7135339_19        WGSGLGFHWSNEHRDRVQALVHMESVVRPVLSWDRFPEVARNIFQALRSDAGEEIVLQKN
K45ctg7152679_7         WGSGLGRHWSNEHRDRVQALIHMESLASCIPSWDLFPEVASNVFQALRSDAGEEMVLKKN
K51ctg5324073_5         WGSGLGFHWSNEHRDRVQALIHMESLASCIPSWDLFPEVASNVFQALRSDAGEEMVLKKN
K35ctg13194577_2        ------------------------------------------------------------
K45ctg7170306_22        ------------------------------------------------------------

Renilla_Luciferase      FFVETMLPSKIMR----------------------------KEIPLVKGGKPDVVQIVRN
K55ctg4472189_6         FFVEKLLPLSIMRKLTDEEMAEYRRPFLEPGESRRPTLTWPREIPVVSDGPQDVVNVVEA
K51ctg5345809_19        FFVEKLLPLAIMRKLTDEEMAEYRRPYMEPGEDRRPTLTWPREIPIVSDGPEDVVKLVEA
K45ctg7135339_19        FFVEKLLPLAIMRKLTDEEMAEYRRPYMEPGEDRRPTLTWPREIPIVSDGPEDVVKLVEA
K45ctg7152679_7         FFVEKLLPLSIMRKLTDEEMAEYRRPFLEPGESRRPTLTWPREIPVVSDGPQDVVNVVEA
K51ctg5324073_5         FFVEKLLPLSIMRKLTDEEMAEYRRPFLEPGESRRPTLTWPREIPVVSDGPQDVVNVVEA
K35ctg13194577_2        FFVEKLLPLSIMRKLTDEEMAEYRRPFLEPGESRRPTLTWPREIPVVSDGPQDVVNVVEA
K45ctg7170306_22        -----------------------------------PTLTWPREIPVVSDGPQDVVNVVEA

Renilla_Luciferase      YNAYLRASDDLPKMFIESDPGFFSNAIVEGAKKFPNTEFVKVKGLHFSQEDAPDEMGKYI
K55ctg4472189_6         YNSWLSESADLPKLYINAEPGFFSPGIKQICAKWPNQKIVTVPGLHFLQEDSPNEIGQAI
K51ctg5345809_19        YHSWLSESDDLPKLYINGEPGFFSPGIKKTCAKWPNQKTVNVPGLHFLQEDSPTEIGQAI
K45ctg7135339_19        YHSWLSESDDLPKLYINGEPGFFSPGIKKTCAKWPNQKTVNVPGLHFLWEDSPTEIGQAI
K45ctg7152679_7         YNSWLSESADLPKLYINAEPGFFSPGIKQICAKWPNQKIV--------------------
K51ctg5324073_5         YNSWLSESADLPKLYINAEPGFFSPGIKQICAKWPNQKIVTVPGLHFLQEDSPNEIGQAI
K35ctg13194577_2        YNSWLSESADLPKLYINAEPGFFSPGIKQICAKWPNQKIVTVPGLHFLQEDSPNEIGQAI
K45ctg7170306_22        YNSWLSESADLPKLYINAEPGFFSPGIKQICAKWPNQKIVTVPGLHFLQEDSPNEIGQAI

Renilla_Luciferase      KSFVERVLKNEQ
K55ctg4472189_6         RTFLQEVY----
K51ctg5345809_19        RTFLEEVYA---
K45ctg7135339_19        RTFLEEVYASN-
K45ctg7152679_7         ------------
K51ctg5324073_5         RTFLQEVY----
K35ctg13194577_2        RTFLQEVYSTK-
K45ctg7170306_22        RTFLQEVYSTK-
```
